# Host Genetic Variation Influences Gene Expression Response to Rhinovirus Infection

**DOI:** 10.1371/journal.pgen.1005111

**Published:** 2015-04-13

**Authors:** Minal Çalışkan, Samuel W. Baker, Yoav Gilad, Carole Ober

**Affiliations:** Department of Human Genetics, The University of Chicago, Chicago, Illinois, United States of America; Georgia Institute of Technology, United States of America

## Abstract

Rhinovirus (RV) is the most prevalent human respiratory virus and is responsible for at least half of all common colds. RV infections may result in a broad spectrum of effects that range from asymptomatic infections to severe lower respiratory illnesses. The basis for inter-individual variation in the response to RV infection is not well understood. In this study, we explored whether host genetic variation is associated with variation in gene expression response to RV infections between individuals. To do so, we obtained genome-wide genotype and gene expression data in uninfected and RV-infected peripheral blood mononuclear cells (PBMCs) from 98 individuals. We mapped local and distant genetic variation that is associated with inter-individual differences in gene expression levels (eQTLs) in both uninfected and RV-infected cells. We focused specifically on response eQTLs (reQTLs), namely, genetic associations with inter-individual variation in gene expression response to RV infection. We identified local reQTLs for 38 genes, including genes with known functions in viral response (*UBA7*, *OAS1*, *IRF5*) and genes that have been associated with immune and RV-related diseases (e.g., *ITGA2*, *MSR1*, *GSTM3*). The putative regulatory regions of genes with reQTLs were enriched for binding sites of virus-activated STAT2, highlighting the role of condition-specific transcription factors in genotype-by-environment interactions. Overall, we suggest that the 38 loci associated with inter-individual variation in gene expression response to RV-infection represent promising candidates for affecting immune and RV-related respiratory diseases.

## Author Summary

Rhinovirus (RV) is the predominant cause of the common cold. However, infections with RV result in a broad spectrum of effects ranging from asymptomatic infections to severe lower respiratory illnesses. We hypothesized that diversity in response to RV-infections is, at least in part, due to variation in the host genome. To address this, we mapped the genetic variations that are associated with gene expression response (reQTLs) to RV-infection in PBMCs. Here, we report local reQTLs for 38 genes including those with known functions in viral response such as *UBA7*, *OAS1*, *IRF5* and those that have been previously associated with immune and RV-related diseases (e.g., *ITGA2*, *MSR1*, *GSTM3*). We also show that reQTL regions are enriched for binding sites of the virus-activated STAT2 transcription factor, suggesting a potential mechanism of action for five of the reQTLs identified. Overall, the reQTLs we identified represent promising candidates to affect individual’s immune response to RV infections and further targeted studies of the reQTL regions might lead to improved control and treatment of RV-associated immune and respiratory diseases.

## Introduction

Rhinovirus (RV) is the most prevalent human respiratory pathogen [1]. It was discovered as the predominant cause of the common cold over 50 years ago [2]. Longitudinal studies indicate that nearly all individuals experience at least one RV infection by two years of age [3]. Each year following, pre-school age children experience six [4] and adults experience two to three [5] RV infections on average. Recent studies have shown that infection with RV results in a broad spectrum of illness severity, ranging from asymptomatic infections to severe lower respiratory illnesses such as bronchiolitis and pneumonia [6]. In addition, RV infections contribute to the morbidity of chronic respiratory illnesses such as asthma, chronic obstructive pulmonary disease (COPD), and cystic fibrosis (CF) [6]. The diversity in response to RV infection is likely attributable to, at least in part, inter-individual variation in the host genome. Indeed, genetic variation in the promoter region of the *IL-10* gene was shown to influence severity of RV illnesses in a small sample of 18 subjects [7] and polymorphisms at the 17q12-q21 asthma locus were associated with both the occurrence and number of RV wheezing illnesses in early life [8]. Beyond these few associations however, the genetic and/or mechanistic basis for the vast inter-individual variation in the response to RV infection is not well understood.

Many cell types are involved in the immune response to RV [9]. Genome-wide gene expression response to RV infection was previously studied in bronchial epithelial cells [10,11] and in nasal epithelial scrapings [12]. While the nasal mucosa is considered the primary site of RV replication [13], RV genomes were found in pericardial fluid, stool, urine, plasma, and serum samples of children with respiratory illnesses, suggesting that systemic infection of RV occurs [14–16]. In addition, RV infection induces cytokine production from monocytes and macrophages without productive viral replication [17], raising the possibility that RV-associated respiratory illnesses may result from virus-induced inflammatory cytokines rather than to cytopathic effects of RV *per se* [18]. To date, however, there have been no genome-wide studies of gene expression response to RV infection in peripheral blood mononuclear cells (PBMCs), or studies characterizing the genetic architecture of inter-individual regulatory variation in gene expression response to RV.

To begin addressing this gap, we have collected and analyzed gene expression data in PBMCs of 98 individuals, before and after RV infection *in vitro*. Our study design allowed us to provide a comprehensive view of the regulatory variation involved in gene expression levels (eQTLs) in uninfected and RV-infected PBMCs. We also identified genetic variations that are specifically associated with differences in gene expression response (reQTLs) to RV infection; these are loci that interact, directly or indirectly, with the infection process and likely include a subset of loci that contribute to the inter-individual variation in the clinical response to RV infection.

## Results

### Rhinovirus infection has a systematic effect on gene expression

**Fig 1:**
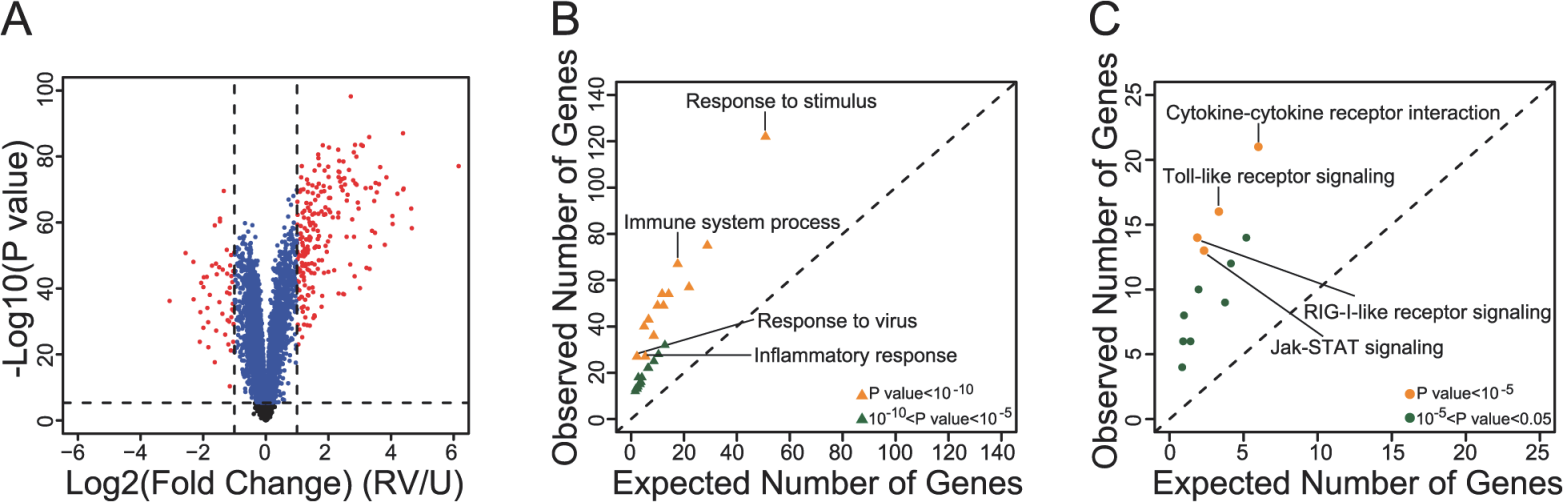
Identification and functional characterization of RV-responsive genes in PBMCs. **(A)** Volcano plot showing the Log2 Fold change (x-axis) and the—Log10 P value (y-axis) of gene expression between uninfected and RV-infected PBMCs. Genes that were not classified as differentially expressed are shown in black. Genes that were significantly differentially expressed (*P*<4.6x10^-6^) but displayed <2-fold change are shown in blue; genes that were both significantly differentially expressed and had ≥2-fold change are shown in red. (**B)** Gene ontology (GO) enrichment results of the genes that were both significantly differentially expressed and had ≥2-fold change (see S3 Table for results including all GO terms). **(C)** Kyoto Encyclopedia of Genes and Genomes (KEGG) pathway enrichment results of the genes that were both significantly differentially expressed and had ≥2-fold change (see S4 Table for results including all KEGG terms).

We obtained genome-wide array-based gene expression data from uninfected and RV-infected PBMCs from 98 unrelated adults (GEO accession number: GSE53543). We detected as expressed 13,881 autosomal probes (targeting 10,893 genes; see Materials and Methods). Across the 196 samples (uninfected and RV-infected paired samples from 98 individuals), gene expression profiles clearly clustered into two major groups by treatment status (by PCA; see Methods; S1 Fig and S2 Fig). We next identified the differentially expressed genes between uninfected and RV-infected PBMCs; of the 10,893 expressed genes, 2,242 were up-regulated and 3,779 were down-regulated in RV-infected compared to uninfected PBMCs (at the Bonferroni corrected significance threshold of *P*<4.60x10^-6^; Fig. 1A, S1 Dataset). We note that with a samples size of 98 individuals we have considerable power to detect differences in gene expression levels. Indeed, the vast majority of effect sizes we detected as significant were minor (Fig. 1A).

We focused, therefore, on the 271 genes that were both significantly differentially expressed and showed at least two-fold difference in expression between uninfected and RV-infected PBMCs. Although a two-fold threshold is arbitrary, it is consistent with the thresholds used in other studies of gene expression response to RV [11,12] and thus provides some measure of consistency with previous reports. In fact, considering our data in the context of other studies we observed a large overlap between the genes that respond to RV in different cell types (86.5% overlap between PBMCs and bronchial epithelial cells [S1 Table] and 73.3% overlap between PBMCs and nasal epithelial scrapings [S2 Table]). Additionally, pathway analysis of the 271 genes revealed enrichment for immune and viral response related pathways, as expected (Fig. 1B, Fig. 1C, S3 Table, S4 Table).

### Mapping eQTLs in uninfected and rhinovirus-infected PBMCs

**Fig 2:**
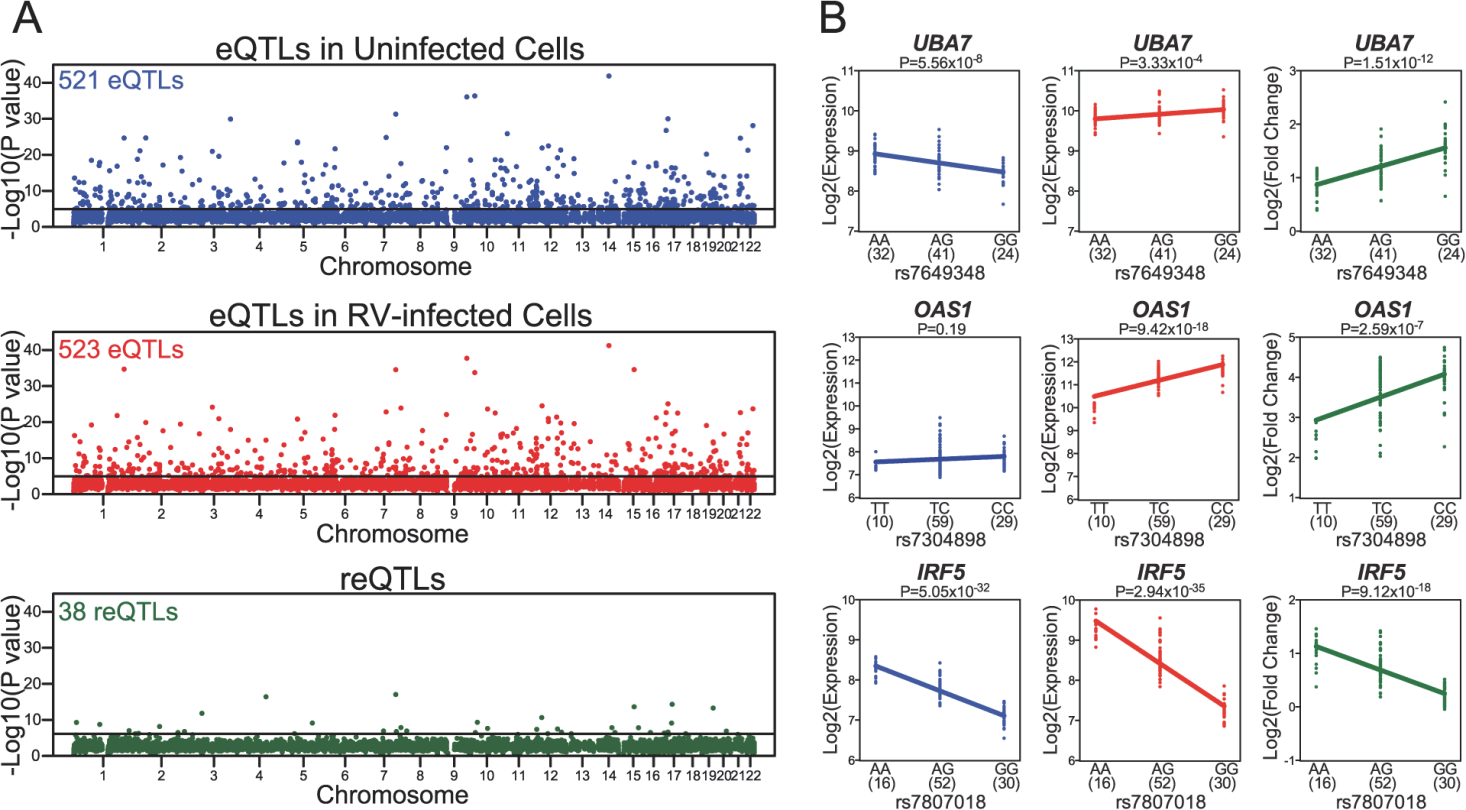
Identification of local eQTLs and reQTLs. **(A)** Manhattan plots of local eQTLs in uninfected cells (in blue), local eQTLs in RV-infected cells (in red), and local reQTLs (in green). Most significant local eQTL or local reQTL P value for each gene (y-axis) is displayed in the order of chromosomal positions of the genes detected as expressed in our study (x-axis). **(B)** Examples of genes with reQTLs. In each plot, genotype at the reQTL is shown on the x-axis and expression level (Log2 Expression) or response in gene expression (Log2 Fold Change) is shown on the y-axis. Sample sizes for each genotype group are shown in parentheses under the x-axis.

We next identified local (putative *cis*) eQTLs in uninfected and RV-infected PBMCs by testing for associations between expression levels and genetic variation at loci within 1 Mb windows from the nearest annotated end of each expressed gene. We performed this analysis separately in uninfected and RV-infected PBMCs, and in both cases we used the SNP with the minimum P value observed for each gene to assess the evidence of an eQTL at that locus. At a false discovery rate (FDR) of 5% based on permutations (which also consider the minimum P value for each gene), we identified 521 genes with local eQTLs in uninfected PBMCs (Fig. 2A, S2 Dataset) and 523 genes with local eQTLs in RV-infected PBMCs (Fig. 2A, S2 Dataset).

For each significant local eQTL-gene expression pair in uninfected and in RV-infected PBMCs, we compared the evidence of eQTL association across conditions (S3 Fig). Pearson correlation of eQTL association P values was 0.84 for the 521 eQTLs in uninfected cells (*P*<10^–15^) and 0.81 for the 523 eQTLs in RV-infected cells (*P*<10^–15^), suggesting that a significant proportion of genetic regulation of gene expression is maintained between uninfected and RV-infected PBMCs.

### Mapping response eQTLs (reQTLs)

We reasoned that SNPs that are specifically associated with gene expression response to RV infection (reQTLs) are likely to have a role in RV-specific response. To identify reQTLs, we tested associations between the expression response (estimated as Log2 fold change in gene expression response to RV infection) and genetic variation in loci that are within 1 Mb of the nearest end of each expressed gene. This analysis revealed local reQTLs for 38 genes at the FDR 5% threshold (Fig. 2A, S2 Dataset). For 25 of the genes with reQTLs, genotypic effect on gene expression at FDR 5% threshold was present only in uninfected or only in RV-infected cells (S2 Dataset). For the remaining 13 genes, genotypic effect on gene expression was present in both uninfected and RV-infected cells at FDR 5% threshold, but the effect size of association was significantly different between conditions (S2 Dataset). Overall, expression levels of genes with reQTLs were slightly higher in the condition where absolute genotypic effect size of the reQTL was larger (S4 Fig). This analysis raises the possibility that slight differences in power to map eQTLs may explain a subset of our observations, but we note that the average difference in expression level is small and is not consistent across all genes with reQTLs.

The 38 genes with reQTLs included those with known functions in viral response, such as *UBA7*, *OAS1*, *IRF5* (Fig. 2B). The protein product of *UBA7* (ubiquitin-like modifier activating enzyme 7) is involved in the activation of a critical antiviral protein ISG15 (interferon-stimulated gene 15) [19,20]. OAS1 (2’-5’-oligoadenylate synthase 1) activates latent RNase L following viral infections and results in degradation of viral RNA [21,22]. Similarly, IRF5 (interferon regulatory factor 5) protein is a direct transducer of virus-mediated signaling [23]. In addition, a subset of the 38 genes with local reQTLs have previously been associated with immune or RV-associated diseases (such as asthma, COPD, and CF; as catalogued by the Genetic Association Database [24]). Specifically, eight genes (*IRF5*, *UBA7*, *TMTC1*, *GSTM3*, *FBN2*, *OAS1*, *PODXL*, *ITGA2*) were previously associated with immune system diseases in at least one study [25–29]. Notably, three genes (*IRF5*, *GSTM3*, *FBN2*) have been associated with asthma [29–31], two (*GSTM3*, *MSR1*) with COPD [32,33] and one (*GSTM3*) with CF [34]. It should be noted, however, that while these observations represent a slight enrichment compared to genome-wide expectations (S5 Fig), enrichment P values were not significant.

In an attempt to fine map the causal reQTLs, we next repeated reQTL mapping for the 38 genes with significant reQTLs using imputed genotype data (see Material and Methods for details of imputation). We compared the most significant reQTL for each gene based on imputed vs. genotyped data (S5 Table). For 16 of the genes, the most significant reQTL based on genotyped data remained the most significant reQTL based on imputed genotype data. However, for 12 of the genes, multiple SNPs had the smallest reQTL P value in the imputed genotypes, suggesting that fine mapping efforts using imputed genotype data in a sample size of 98 individuals is not sufficient to identify the causal reQTL.

### Enrichment of STAT2 binding sites among reQTL loci

**Fig 3:**
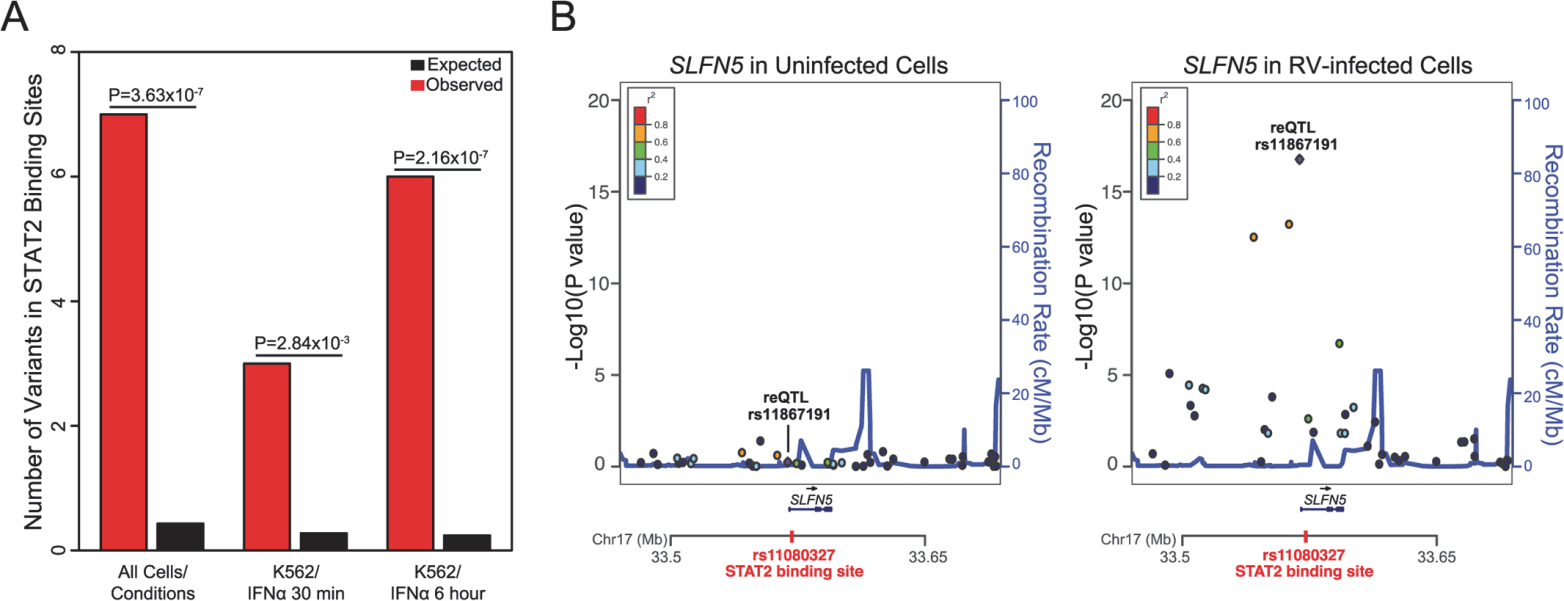
STAT2 binding sites are enriched among reQTL loci. **(A)** Observed and expected numbers of variants that lie within STAT2 binding sites i) across all human cell types/conditions available in ENCODE ChIP-Seq data, ii) in IFNα-30 minute treated human K562 cell line, iii) in IFNα-6 hour treated human K562 cell line. **(B)** Regional eQTL association plots of *SLFN5* in uninfected and RV-infected cells. See S6 Fig for regional eQTL association plots of the remaining four genes with significant reQTL loci SNPs that reside in STAT2 binding sites. In all five cases, the eQTL association was significant only in RV-infected cells.

Previous studies have suggested that reQTLs can often be found within the binding sites of transcription factors that have different levels of activity between the conditions being tested [35,36]. We examined this in our data by considering the overlap between known protein binding sites (based on ENCODE ChIP-Seq data [37]) and the 38 reQTLs identified in our study. This analysis is challenging because of the uncertainty in identifying the causal reQTL association. We therefore considered for this analysis the reQTLs and all other common SNPs in high LD with the reQTLs (r^2^>0.8 and MAF>0.1 based on 1000 Genomes Phase I European Population) as input SNPs. We then extracted 1,000 control SNPs (matched based on MAF, gene density, distance to nearest gene, number of SNPs in LD) per each input SNP and examined the overlap between protein binding sites and each unique SNP in the input and control SNP lists. This analysis revealed that STAT2 was the most substantially enriched transcription factor (16.30 fold; *P*<10^–6^; S6 Table and Fig. 3A) with binding sites overlapping reQTL loci SNPs relative to the control SNPs (see Materials and Methods for details of enrichment analysis).

STAT2 is a critical regulator of anti-viral response [38]. In our study, *STAT2* gene expression increased by 3.68 fold (S1 Dataset; *P*<10^–77^) in response to RV infection. Based on this observation, we hypothesized that reQTL loci SNPs that lie within STAT2 binding sites should have stronger regulatory effects on gene expression in RV-infected cells relative to uninfected cells. As expected, for all five targets of STAT2 among our reQTL regions, the eQTL effects on gene expression were stronger in RV-infected cells compared to uninfected cells (Fig. 3B and S6 Fig). Consistent with this observation is that all the STAT2 ChIP-Seq signals (based on ENCODE ChIP-Seq data) in our reQTL loci were identified in the IFNα treated human K562 cell line (S7 Table and Fig. 3A). Additionally, our results imply that the SNPs residing in STAT2 binding sites are more likely to be the causal SNP in the reQTL loci of *EXOSC9*, *SLFN5*, *PRR24*, *OAS1*, and *ARL5B*. In fact, causality of the reQTL of *SLFN5* gene, rs11080327, was recently demonstrated by luciferase reporter assays [35].

### Little evidence for trans reQTLs

Lastly, we searched for distant (putatively *trans*) eQTLs and reQTLs by testing associations between SNP-gene combinations for which the SNP distance from the nearest end of the gene was more than 5 Mb. We used the minimum P value observed per gene to assess the significance of the distant eQTL or reQTL. We identified 11 and 15 genes with distant eQTLs in uninfected and in RV-infected PBMCs, respectively (at an FDR 5% based on permutations; S3 Dataset). We found no direct evidence for distant reQTLs when we considered genetic associations with the expression response to infection (S3 Dataset), yet 6 of the *trans* eQTLs were specific to either the infected or uninfected cells. Because *trans* eQTL findings based on microarray expression data tend to suffer more from a high degree of false-positives, partly due to cross-hybridizations, we considered carefully each significant finding. For each gene that was putatively associated with a *trans* eQTL, we re-mapped each of its probes within 2 Mb of the *trans* eQTL using SHRiMP [39], as previously described [40]. We discarded all *trans* eQTLs whose associated trans-probe mapped in its vicinity. After exclusions, only 4 and 6 genes with distant eQTLs in uninfected and RV-infected cells, respectively, remained; 3 of them were common to both conditions (S8 Table). Thus, 4 *trans* eQTLs may be reQTLs, yet considerations of incomplete power for detecting *trans* eQTLs in our study call for caution in this interpretation.

## Discussion

Mapping of condition-specific or response eQTLs in a genome-wide level is an emerging area of research with the promise of understanding biology of infectious diseases [41,42], response to pharmaceutical treatment [36], and inter-individual variation in immune response more broadly [35,43]. Here, we report 38 local reQTLs that are associated with inter-individual variation in gene expression response to RV infection. These loci are likely to interact, directly or indirectly, with the infection process. Because infection with rhinovirus causes significant changes in regulatory activity of the identified reQTLs, these variants represent promising candidates for susceptibility and response to RV infections *in vivo*. In support of this reasoning, we pointed to eight genes with RV-reQTLs that have been previously associated with immune diseases, and four that were previously associated with respiratory diseases (either asthma, COPD, or CF).

We note that while the numbers of disease-associated genes among genes with reQTLs were greater than the genome-wide expectations, none of the enrichment P values were significant. That said, it is important to emphasize that RV infections, in general, are thought to affect the morbidity of the chronic respiratory illnesses rather than the risk of developing the disease [6]. Therefore, it is possible that the reQTLs identified here are more likely to influence the severity of the respiratory illnesses rather than the occurrence, which has been the focus of the majority of the association studies performed thus far. Similarly, risk of developing the common cold might be more directly influenced by the reQTLs identified here because RV is causally associated with development of the common cold. However, to our knowledge, there have been no association studies on the frequency or severity of common colds.

It is also possible that at least some of the reQTLs identified here are functional in response to a broader range of stimuli, including other pathogens. In fact, six (*SLFN5*, *ARL5B*, *SPTLC2*, *IRF5*, *ADCY3*, *CCDC146*) of the 38 genes implicated in our reQTL mapping were also identified in a recent reQTL mapping study for *Escherichia coli* lipopolysaccharide, influenza, and interferon-β in dendritic cells [35]. Further comparative studies of reQTLs will be necessary to disentangle stimulus-specific and shared reQTLs. We also note that some of the reQTLs identified in our study may be confounded due to cell type heterogeneity in PBMCs. In our study, we were unable to count cell subsets of PBMCs before and after RV infection. If for instance, cell subset proportions of PBMCs change in response to RV infection, statistical power to identify cell-type specific eQTLs may differ between uninfected and RV-infected PBMCs and this may potentially lead to identification of cell-type specific eQTLs as reQTLs. Similarly, it is possible that cell type heterogeneity in PBMCs may mask identification of cell-type specific reQTLs, especially those that are specific to rare cell subsets of PBMCs. However, we also appreciate the fact that gene expression response in a complex system of interacting cells as in PBMCs might be more relevant to true physiological responses than those observed in purified cell subsets.

The enrichment of STAT2 binding sites among reQTL regions highlights the role of condition-specific transcription factors in gene-by-environment interactions. Our results suggest that transcription factor activation upon RV-infection reveals SNPs with regulatory activity that could not be identified in uninfected PBMCs. This hypothesis is also supported by the fact that all STAT2 ChIP-Seq signals in our reQTL regions were identified in the IFNα treated human K562 cell line. IFNα is involved in the innate immune response against viral infections and our results therefore suggest that RV infection may activate STAT2 through the IFNα signaling pathway.

In conclusion, we have provided a comprehensive genome-wide view of host genetic variation that is associated with gene expression response to rhinovirus infection. The reQTLs identified here are promising candidates to influence both the frequency and the severity of RV related respiratory illnesses. Additionally, our results contribute to the field of genotype-by-environment interactions and might further help to disentangle stimulus-specific and shared reQTLs.

## Materials and Methods

### Ethics statement

One hundred unrelated adult volunteers (49 males and 51 females; age range 19–60) were recruited between July and November 2011 to study the genotype-specific effects of RV infection on gene expression patterns in PBMCs. Informed written consent was obtained from each study participant. This study was approved by the Institutional Review Board at the University of Chicago.

### Sample collection and experimental design

Twenty ml of blood was drawn from each participant. PBMCs were isolated from whole blood samples by Ficoll-Paque separation [44]. From each subject, 4x10^6^ PBMCs were treated with media alone for 24 hours and 4x10^6^ PBMCs were treated with media containing RV16 for 24 hours. The multiplicity of infection was 10 plaque-forming units per cell.

### DNA extraction and genome-wide genotyping

DNA was extracted on the day of sample collection using QIAamp DNA Blood Mini Kit; concentrations were measured on a Nanodrop ND-100 Spectrophotometer. Genotyping of 100 individuals was performed using the Axiom Genome Wide Human Array Plate–CEU, which interrogates 669,059 SNPs. Genotype calls were extracted from the raw data using Affymetrix Power Tools software. One individual with genotype call rate less than 97% and one individual that failed Affymetrix gender call were excluded from all further analyses. After exclusions, the sample size was 98 (49 males and 49 females; age range 19–60). Following quality-control checks (Hardy-Weinberg equilibrium *P*>10^–6^, MAF>0.1, SNP call rate>95%), 382,855 SNPs were retained and 373,312 autosomal SNPs with unique SNP identifiers were used in subsequent analyses.

Global proportions of European, Asian, and African ancestry in our samples were estimated by using the program ADMIXTURE [45] and assuming 3 ancestral populations (K = 3) (S7 Fig). Subjects from the Phase 3 HapMap CEU (Utah residents with Northern and Western European ancestry from the CEPH collection), CHB (Han Chinese in Beijing, China), JPT (Japanese in Tokyo, Japan), and YRI (Yoruba in Ibadan, Nigeria) were included as reference populations. 85 of the individuals had over 80% European ancestry, six of them had over 80% Asian ancestry and the remaining seven individuals had relatively more mixed ancestry fractions. Ancestry estimates were taken into account in further analyses to correct for the potential effects of ancestry on gene expression profiles.

### Genotype imputation

373,312 SNPs that passed the genotyping quality-control checks were used to perform the pre-phasing of the chromosomes using SHAPEIT (v2.r790) [46]. Imputation was performed using IMPUTE2 (2.3.1) [47] over genomic regions of 5 Mb, as recommended. For both pre-phasing and imputation, 1000 Genomes Phase 3 data were used as the reference panel.

78,091,231 autosomal variant sites were imputed across 98 individuals. After quality-control checks (Hardy-Weinberg equilibrium *P*>10^–6^, MAF>0.1, SNP call rate>95%), 3,722,989 of the imputed variants were retained.

### RNA extraction and gene expression profiling

Total RNA was extracted after 24-hour incubation, using the RNeasy Plus Mini Kit; concentrations were measured on a Nanodrop ND-100 Spectrophotometer and quality was assessed using an Agilent 2100 Bioanalyzer. Genome wide gene expression profiling of uninfected and RV-infected PBMCs was obtained using Illumina HumanHT-12 v4 Expression BeadChip arrays, which targets 47,305 probes. The cDNA synthesis, labeling, and hybridization of RNA to the microarrays were performed at the University of Chicago Functional Genomics Core.

The process of quality-control checks (probes that mapped to unique Ensembl gene IDs, probes that did not contain any HapMap SNPs with MAF>0.01 in the CEU population, probes that targeted autosomal chromosomes) resulted in retention of 26,440 probes. Among those, 13,881 probes that were detected as expressed in PBMCs (detection *P*<0.05 in at least 25% of the samples) were used in subsequent analyses. Low-level microarray analyses were performed in R (http://www.R-project.org), using the Bioconductor software package lumi [48]. Probe intensity estimates were log_2_-transformed and rank-invariate normalized.

Linear models were used to test the relationship between each known covariate (gender, virus batch, processing day, chip number, PBMC count, age, and ADMIXTURE’s [45] Q estimates (the ancestry fractions)) and the principal components that explain at least 5% of the total variance in the gene expression data. (S1 Fig). Processing day was significantly associated with the Principal Component 2 and hence it was included as an “adjustment variable” when performing SVA [49]. SVA analysis yielded no significant surrogate variables when “adjustment variable” of processing day was used with “variable of interest” of treatment. The effects of processing day were regressed out of the gene expression data prior to further analyses (S2 Fig).

Median probe intensity estimates per gene were calculated as the expression levels for 10,893 genes and used in all further analyses.

### Identification of differentially expressed genes between uninfected and RV-infected cells

Differentially expressed genes between uninfected PBMCs and RV-infected PBMCs were identified using a paired t-test in R statistical environment. Significance was calculated using the Bonferroni correction at α = 0.05 (*P*<4.60x10^-6^).

### Gene ontology, KEGG pathway enrichment analyses

The DAVID bioinformatics database [50] was used to test for enrichment of Gene Ontology (GO) categories (BP_ALL) and Kyoto Encyclopedia of Genes and Genomes (KEGG) pathways among the genes that were both statistically differentially expressed and had ≥2-fold change in response to RV infection. 10,893 genes that were detected as expressed in our data were used as the background set in all enrichment analyses. Enrichment P values were calculated using a modified Fisher Exact test (EASE Score).

### Genetic mapping of gene expression and gene expression response

Associations between local (defined as SNP-gene pairs within 1 Mb window from the nearest end of the gene) and distant (defined as SNP-gene pairs for which the SNP distance from the nearest end of the gene was more than 5 Mb) SNPs and gene expression in uninfected and in RV-infected cells were tested using linear regressions with additive genotype effects and taking ancestry estimates into account. Similarly, associations between local and distant SNPs and gene expression response (Log2 fold change in gene expression response to RV infection) were tested using linear regressions with additive genotype effects and taking ancestry estimates into account. All the analyses were performed as implemented in “linear” model of the Matrix eQTL package [51]. The minimum P value observed for each gene was recorded and used as the evidence of eQTL or reQTL association.

To estimate the FDR, each of the phenotype data (gene expression or gene expression response) were shuffled 100 times and linear regressions were repeated using each set of permuted data. The minimum P value for each gene was recorded and used as the empirical null distribution. Permutation-based FDR was calculated using fdrci package [52] in R.

### Analysis of gene-disease associations

Genes previously reported to be associated with immune diseases (Disease Class) and RV-related respiratory illnesses (asthma, COPD, CF) (Disease Terms) were downloaded from the Genetic Association Database (GADCDC data as of 08/18/2014). Enrichment P values were calculated using Fisher's exact test.

### ChIP-Seq enrichment analysis

SNP ‒ human ChIP-Seq (based on ENCODE data [37]) annotation data were downloaded from HaploReg v2 [53] in December 2014. For each ChIP annotation across all available cell types and conditions, the frequency of overlap between unique reQTL loci SNPs was compared with the frequency of overlap with a set of unique control SNPs.

reQTL loci SNPs included the list of reQTLs and all other common SNPs in high LD with the reQTLs; r^2^>0.8 and MAF>0.1 based on 1000 Genomes Phase I European Population. 1,000 control SNPs (matched based on MAF [±5% point], gene density [±50%], distance to nearest gene [±50%], number of SNPs in LD [±50% using r^2^ 0.5]) per each input SNP were pulled using the SNPsnap webserver [54]. Binomial P values and fold-enrichments were calculated for proteins with at least two ChIP-Seq signal overlapping with the reQTL loci SNPs.

For STAT2 ChIP annotation, the frequency of overlap between reQTL loci SNPs and the matching control SNPs was additionally compared within IFNα-30 minute and IFNα-6 hour treated human K562 cell line.

### Quality control check of trans eQTLs

To minimize the false-positive *trans* eQTL findings, each probe targeting the genes with significant *trans* eQTLs was re-mapped using SHRIMP [39] with relaxed mapping setting that was previously described; match score of 10, mismatch score of 0, gap open penalty of −250, gap extension penalty of −100, and minimal Smith-Waterman score of 30% [40]. *Trans* eQTLs whose associated trans-probe mapped in its vicinity were excluded.

## Supporting Information

S1 Fig Clustering of gene expression data. **(A)** Heatmap clustering, and **(B)** Principal component plot of 13,881 probes after log_2_-transformation and rank-invariate normalization. **(C)** P values from linear models testing the relationship between each known variable (potential covariates and variable of interest) and the principal components that explain at least 5% of the total variance in the gene expression data are shown. P values that are significant (after Bonferroni correction at α = 0.05) are highlighted in red.
(PDF)

S2 Fig Clustering of gene expression data after regressing out processing day. **(A)** Heatmap clustering, and **(B)** Principal component plot of 13,881 probes after log_2_-transformation, rank-invariate normalization, and regressing out processing day. **(C)** P values from linear models testing the relationship between each known variable (potential covariates and variable of interest) and the principal components that explain at least 5% of the total variance in the gene expression data are shown. P values that are significant (after Bonferroni correction at α = 0.05) are highlighted in red.
(PDF)

S3 Fig Comparisons of the evidence of eQTL association across conditions. **(A)** For 521 significant local eQTL-gene expression pair in uninfected cells, P values in uninfected cells are shown on the x-axis and P values in RV-infected cells are shown on the y-axis. Pearson correlation of eQTL association P values was 0.84 (*P*<2.2x10^-16^). **(B)** For 523 significant local eQTL-gene expression pair in RV-infected cells, P values in RV-infected cells are shown on the x-axis and P values in uninfected cells are shown on the y-axis. Pearson correlation of eQTL association P values was 0.81 (*P*<2.2x10^-16^). **(C)** For 521 significant local eQTL-gene expression pair in uninfected cells, Beta (effect size estimate) values in uninfected cells are shown on the x-axis and Beta values in RV-infected cells are shown on the y-axis. Pearson correlation of Beta values was 0.94 (*P*<2.2x10^-16^). **(D)** For 523 significant local eQTL-gene expression pair in RV-infected cells, Beta values in RV-infected cells are shown on the x-axis and Beta values in uninfected cells are shown on the y-axis. Pearson correlation of Beta values was 0.93 (*P*<2.2x10^-16^).
(PDF)

S4 Fig Expression levels of genes with reQTLs in the condition where absolute genotypic effect size of the reQTL was larger vs. in the condition where absolute genotypic effect size of the reQTL was smaller. (PDF)

S5 Fig Percentage of disease associated genes. Percentage of genes associated with **(A)** Immune diseases **(B)** Asthma **(C)** Chronic Obstructive Pulmonary Disease (COPD) **(D)** Cystic Fibrosis (CF). In each panel, ‘all genes’ category (in black) refers to 10,893 genes detected as expressed and ‘genes with reQTLs’ category (in green) refers to 38 genes that had a significant local reQTL in our study. Enrichment P values (Fisher’s exact test) were 0.17, 0.17, 0.23, and 0.12, respectively.
(PDF)

S6 Fig Regional eQTL association plots of genes with reQTL loci variants that reside in STAT2 binding sites. (PDF)

S7 Fig Global proportions of European, Asian, and African ancestry in our study subjects were estimated using ADMIXTURE, assuming 3 ancestral populations. Subjects from the Phase 3 HapMap CEU (Utah residents with Northern and Western European ancestry from the CEPH collection), CHB (Han Chinese in Beijing, China), JPT (Japanese in Tokyo, Japan), and YRI (Yoruba in Ibadan, Nigeria) were included as reference.
(PDF)

S1 Table Overlap between RV-responsive genes in bronchial epithelial cells (BECs) and in PBMCs. Among the genes that were targeted in both studies, 86.5% (32 out of 37) with ≥2-fold increase in response to RV infection in BECs also showed ≥2-fold increase in response to RV infection in PBMCs.
(PDF)

S2 Table Overlap between RV-responsive genes in nasal epithelial scrapings (NESs) and in PBMCs. Among the genes that were targeted in both studies, 73.3% (22 out of 30) with ≥2-fold increase in response to RV infection in NESs also showed ≥2-fold increase in response to RV infection in PBMCs.
(PDF)

S3 Table Gene ontology (GO) enrichment analysis for the 271 genes that were both statistically differentially expressed and had a fold change of ≥2 in response to RV infection. Enriched GO categories at P value cut-off of 10^–5^ are shown.
(PDF)

S4 Table Kyoto Encyclopedia of Genes and Genomes (KEGG) pathway enrichment analysis for the 271 genes that were both statistically differentially expressed and had a fold change of ≥2 in response to RV infection. Enriched KEGG pathways at P value cut-off of 0.05 are shown.
(PDF)

S5 Table Comparison of most significant reQTL based on imputed and genotyped data. (PDF)

S6 Table ChIP-Seq enrichment results of input SNPs relative to the background SNPs based on all available human cell types and treatment conditions in the ENCODE data. (PDF)

S7 Table reQTL loci variants that overlap with STAT2 ChIP-Seq annotation. LD (r^2^) is based on 1000 Genomes Phase 1 EUR population.
(PDF)

S8 Table Quality control check of genome-wide significant distant eQTLs (A) in uninfected cells (B) in RV-infected cells. (PDF)

S1 Dataset Summary of the results of differential expression analysis. (XLSX)

S2 Dataset Summary of the results of local eQTL and reQTL mapping. (XLSX)

S3 Dataset Summary of the results of distant eQTL and reQTL mapping. (XLSX)

## References

[1] Arruda E, Pitkaranta A, Witek TJ Jr., Doyle CA, Hayden FG (1997) Frequency and natural history of rhinovirus infections in adults during autumn. J Clin Microbiol 35: 2864–2868. 9350748 10.1128/jcm.35.11.2864-2868.1997 PMC230076

[2] Andrewes CH, Chaproniere DM, Gompels AE, Pereira HG, Roden AT (1953) Propagation of common-cold virus in tissue cultures. Lancet 265: 546–547. 13097995 10.1016/s0140-6736(53)90279-7

[3] Fox JP, Cooney MK, Hall CE (1975) The Seattle virus watch. V. Epidemiologic observations of rhinovirus infections, 1965–1969, in families with young children. Am J Epidemiol 101: 122–143. 164769 10.1093/oxfordjournals.aje.a112078

[4] Winther B, Hayden FG, Hendley JO (2006) Picornavirus infections in children diagnosed by RT-PCR during longitudinal surveillance with weekly sampling: Association with symptomatic illness and effect of season. J Med Virol 78: 644–650. 16555289 10.1002/jmv.20588

[5] Winther B (2011) Rhinovirus infections in the upper airway. Proc Am Thorac Soc 8: 79–89. 10.1513/pats.201006-039RN 21364225

[6] Gern JE (2010) The ABCs of rhinoviruses, wheezing, and asthma. J Virol 84: 7418–7426. 10.1128/JVI.02290-09 20375160 PMC2897627

[7] Helminen M, Nuolivirta K, Virta M, Halkosalo A, Korppi M, et al (2008) IL-10 gene polymorphism at -1082 A/G is associated with severe rhinovirus bronchiolitis in infants. Pediatr Pulmonol 43: 391–395. 10.1002/ppul.20793 18286551

[8] Caliskan M, Bochkov YA, Kreiner-Moller E, Bonnelykke K, Stein MM, et al (2013) Rhinovirus wheezing illness and genetic risk of childhood-onset asthma. N Engl J Med 368: 1398–1407. 10.1056/NEJMoa1211592 23534543 PMC3755952

[9] Kelly JT, Busse WW (2008) Host immune responses to rhinovirus: mechanisms in asthma. J Allergy Clin Immunol 122: 671–682; quiz 683–674. 10.1016/j.jaci.2008.08.013 19014757 PMC3927944

[10] Bochkov YA, Hanson KM, Keles S, Brockman-Schneider RA, Jarjour NN, et al (2010) Rhinovirus-induced modulation of gene expression in bronchial epithelial cells from subjects with asthma. Mucosal Immunol 3: 69–80. 10.1038/mi.2009.109 19710636 PMC2884103

[11] Chen Y, Hamati E, Lee PK, Lee WM, Wachi S, et al (2006) Rhinovirus induces airway epithelial gene expression through double-stranded RNA and IFN-dependent pathways. Am J Respir Cell Mol Biol 34: 192–203. 16210696 10.1165/rcmb.2004-0417OC PMC2644182

[12] Proud D, Turner RB, Winther B, Wiehler S, Tiesman JP, et al (2008) Gene expression profiles during in vivo human rhinovirus infection: insights into the host response. Am J Respir Crit Care Med 178: 962–968. 10.1164/rccm.200805-670OC 18658112

[13] Arruda E, Boyle TR, Winther B, Pevear DC, Gwaltney JM Jr., et al (1995) Localization of human rhinovirus replication in the upper respiratory tract by in situ hybridization. J Infect Dis 171: 1329–1333. 7751712 10.1093/infdis/171.5.1329

[14] Broberg E, Niemela J, Lahti E, Hyypia T, Ruuskanen O, et al (2011) Human rhinovirus C—associated severe pneumonia in a neonate. J Clin Virol 51: 79–82. 10.1016/j.jcv.2011.01.018 21342784 PMC7172304

[15] Fuji N, Suzuki A, Lupisan S, Sombrero L, Galang H, et al (2011) Detection of human rhinovirus C viral genome in blood among children with severe respiratory infections in the Philippines. PLoS One 6: e27247 10.1371/journal.pone.0027247 22087272 PMC3210775

[16] Tapparel C, L'Huillier AG, Rougemont AL, Beghetti M, Barazzone-Argiroffo C, et al (2009) Pneumonia and pericarditis in a child with HRV-C infection: a case report. J Clin Virol 45: 157–160. 10.1016/j.jcv.2009.03.014 19427260 PMC7108322

[17] Gern JE, Vrtis R, Kelly EA, Dick EC, Busse WW (1996) Rhinovirus produces nonspecific activation of lymphocytes through a monocyte-dependent mechanism. J Immunol 157: 1605–1612. 8759745

[18] Yamaya M, Sasaki H (2003) Rhinovirus and asthma. Viral Immunol 16: 99–109. 12828863 10.1089/088282403322017857

[19] Lai C, Struckhoff JJ, Schneider J, Martinez-Sobrido L, Wolff T, et al (2009) Mice lacking the ISG15 E1 enzyme UbE1L demonstrate increased susceptibility to both mouse-adapted and non-mouse-adapted influenza B virus infection. J Virol 83: 1147–1151. 10.1128/JVI.00105-08 19004958 PMC2612374

[20] Yuan W, Krug RM (2001) Influenza B virus NS1 protein inhibits conjugation of the interferon (IFN)-induced ubiquitin-like ISG15 protein. EMBO J 20: 362–371. 11157743 10.1093/emboj/20.3.362 PMC133459

[21] Malathi K, Paranjape JM, Bulanova E, Shim M, Guenther-Johnson JM, et al (2005) A transcriptional signaling pathway in the IFN system mediated by 2'-5'-oligoadenylate activation of RNase L. Proc Natl Acad Sci U S A 102: 14533–14538. 16203993 10.1073/pnas.0507551102 PMC1239948

[22] Silverman RH (2007) Viral encounters with 2',5'-oligoadenylate synthetase and RNase L during the interferon antiviral response. J Virol 81: 12720–12729. 17804500 10.1128/JVI.01471-07 PMC2169107

[23] Barnes BJ, Moore PA, Pitha PM (2001) Virus-specific activation of a novel interferon regulatory factor, IRF-5, results in the induction of distinct interferon alpha genes. J Biol Chem 276: 23382–23390. 11303025 10.1074/jbc.M101216200

[24] Becker KG, Barnes KC, Bright TJ, Wang SA (2004) The genetic association database. Nat Genet 36: 431–432. 15118671 10.1038/ng0504-431

[25] Fedetz M, Matesanz F, Caro-Maldonado A, Fernandez O, Tamayo JA, et al (2006) OAS1 gene haplotype confers susceptibility to multiple sclerosis. Tissue Antigens 68: 446–449. 17092260 10.1111/j.1399-0039.2006.00694.x

[26] Litonjua AA, Lasky-Su J, Schneiter K, Tantisira KG, Lazarus R, et al (2008) ARG1 is a novel bronchodilator response gene: screening and replication in four asthma cohorts. Am J Respir Crit Care Med 178: 688–694. 10.1164/rccm.200709-1363OC 18617639 PMC2556451

[27] Morgan AR, Han DY, Lam WJ, Fraser AG, Ferguson LR (2010) Association analysis of 3p21 with Crohn's disease in a New Zealand population. Hum Immunol 71: 602–609. 10.1016/j.humimm.2010.03.003 20307617

[28] Sigurdsson S, Nordmark G, Goring HH, Lindroos K, Wiman AC, et al (2005) Polymorphisms in the tyrosine kinase 2 and interferon regulatory factor 5 genes are associated with systemic lupus erythematosus. Am J Hum Genet 76: 528–537. 15657875 10.1086/428480 PMC1196404

[29] Wikman H, Piirila P, Rosenberg C, Luukkonen R, Kaaria K, et al (2002) N-Acetyltransferase genotypes as modifiers of diisocyanate exposure-associated asthma risk. Pharmacogenetics 12: 227–233. 11927838 10.1097/00008571-200204000-00007

[30] Choudhry S, Taub M, Mei R, Rodriguez-Santana J, Rodriguez-Cintron W, et al (2008) Genome-wide screen for asthma in Puerto Ricans: evidence for association with 5q23 region. Hum Genet 123: 455–468. 10.1007/s00439-008-0495-7 18401594 PMC2664533

[31] Janssen R, Bont L, Siezen CL, Hodemaekers HM, Ermers MJ, et al (2007) Genetic susceptibility to respiratory syncytial virus bronchiolitis is predominantly associated with innate immune genes. J Infect Dis 196: 826–834. 17703412 10.1086/520886

[32] Ohar JA, Hamilton RF Jr., Zheng S, Sadeghnejad A, Sterling DA, et al (2010) COPD is associated with a macrophage scavenger receptor-1 gene sequence variation. Chest 137: 1098–1107. 10.1378/chest.09-1655 20081102 PMC2862400

[33] Young RP, Hopkins RJ, Hay BA, Epton MJ, Mills GD, et al (2009) A gene-based risk score for lung cancer susceptibility in smokers and ex-smokers. Postgrad Med J 85: 515–524. 10.1136/pgmj.2008.077107 19789190

[34] Flamant C, Henrion-Caude A, Boelle PY, Bremont F, Brouard J, et al (2004) Glutathione-S-transferase M1, M3, P1 and T1 polymorphisms and severity of lung disease in children with cystic fibrosis. Pharmacogenetics 14: 295–301. 15115915 10.1097/00008571-200405000-00004

[35] Lee MN, Ye C, Villani AC, Raj T, Li W, et al (2014) Common genetic variants modulate pathogen-sensing responses in human dendritic cells. Science 343: 1246980 10.1126/science.1246980 24604203 PMC4124741

[36] Maranville JC, Luca F, Richards AL, Wen X, Witonsky DB, et al (2011) Interactions between glucocorticoid treatment and cis-regulatory polymorphisms contribute to cellular response phenotypes. PLoS Genet 7: e1002162 10.1371/journal.pgen.1002162 21750684 PMC3131293

[37] Consortium EP, Bernstein BE, Birney E, Dunham I, Green ED, et al (2012) An integrated encyclopedia of DNA elements in the human genome. Nature 489: 57–74. 10.1038/nature11247 22955616 PMC3439153

[38] Park C, Li S, Cha E, Schindler C (2000) Immune response in Stat2 knockout mice. Immunity 13: 795–804. 11163195 10.1016/s1074-7613(00)00077-7

[39] Rumble SM, Lacroute P, Dalca AV, Fiume M, Sidow A, et al (2009) SHRiMP: accurate mapping of short color-space reads. PLoS Comput Biol 5: e1000386 10.1371/journal.pcbi.1000386 19461883 PMC2678294

[40] Fehrmann RS, Jansen RC, Veldink JH, Westra HJ, Arends D, et al (2011) Trans-eQTLs reveal that independent genetic variants associated with a complex phenotype converge on intermediate genes, with a major role for the HLA. PLoS Genet 7: e1002197 10.1371/journal.pgen.1002197 21829388 PMC3150446

[41] Barreiro LB, Tailleux L, Pai AA, Gicquel B, Marioni JC, et al (2012) Deciphering the genetic architecture of variation in the immune response to Mycobacterium tuberculosis infection. Proc Natl Acad Sci U S A 109: 1204–1209. 10.1073/pnas.1115761109 22233810 PMC3268270

[42] Idaghdour Y, Quinlan J, Goulet JP, Berghout J, Gbeha E, et al (2012) Evidence for additive and interaction effects of host genotype and infection in malaria. Proc Natl Acad Sci U S A 109: 16786–16793. 10.1073/pnas.1204945109 22949651 PMC3479498

[43] Fairfax BP, Humburg P, Makino S, Naranbhai V, Wong D, et al (2014) Innate immune activity conditions the effect of regulatory variants upon monocyte gene expression. Science 343: 1246949 10.1126/science.1246949 24604202 PMC4064786

[44] Fuss IJ, Kanof ME, Smith PD, Zola H (2009) Isolation of whole mononuclear cells from peripheral blood and cord blood. Curr Protoc Immunol Chapter 7: Unit7 1. 10.1002/0471142735.im0701s85 19347849

[45] Alexander DH, Novembre J, Lange K (2009) Fast model-based estimation of ancestry in unrelated individuals. Genome Res 19: 1655–1664. 10.1101/gr.094052.109 19648217 PMC2752134

[46] Delaneau O, Marchini J, Zagury JF (2012) A linear complexity phasing method for thousands of genomes. Nat Methods 9: 179–181. 10.1038/nmeth.1785 22138821

[47] Howie BN, Donnelly P, Marchini J (2009) A flexible and accurate genotype imputation method for the next generation of genome-wide association studies. PLoS Genet 5: e1000529 10.1371/journal.pgen.1000529 19543373 PMC2689936

[48] Du P, Kibbe WA, Lin SM (2008) lumi: a pipeline for processing Illumina microarray. Bioinformatics 24: 1547–1548. 10.1093/bioinformatics/btn224 18467348

[49] Leek JT, Johnson WE, Parker HS, Jaffe AE, Storey JD (2012) The sva package for removing batch effects and other unwanted variation in high-throughput experiments. Bioinformatics 28: 882–883. 10.1093/bioinformatics/bts034 22257669 PMC3307112

[50] Huang da W, Sherman BT, Lempicki RA (2009) Systematic and integrative analysis of large gene lists using DAVID bioinformatics resources. Nat Protoc 4: 44–57. 10.1038/nprot.2008.211 19131956

[51] Shabalin AA (2012) Matrix eQTL: ultra fast eQTL analysis via large matrix operations. Bioinformatics 28: 1353–1358. 10.1093/bioinformatics/bts163 22492648 PMC3348564

[52] Millstein J (2013) fdrci: Permutation-based FDR Point and Confidence Interval Estimation. R package version 20. 10.3389/fgene.2013.00179 PMC3775454 24062767

[53] Ward LD, Kellis M (2012) HaploReg: a resource for exploring chromatin states, conservation, and regulatory motif alterations within sets of genetically linked variants. Nucleic Acids Res 40: D930–934. 10.1093/nar/gkr917 22064851 PMC3245002

[54] Pers TH, Timshel P, Hirschhorn JN (2014) SNPsnap: a Web-based tool for identification and annotation of matched SNPs. Bioinformatics. 10.1093/bioinformatics/btu655 PMC4308663 25316677

