# Supplementary material for: Host Genetic Variation Influences Gene Expression Response to Rhinovirus Infection
Source: PLoS Genet. 2015 Apr 13;11(4):e1005111. doi: 10.1371/journal.pgen.1005111 (PMC4395341; doi:10.1371/journal.pgen.1005111)
Supplement: S1 Fig — (A) Heatmap clustering, and (B) Principal component plot of 13,881 probes after log2-transformation and rank-invariate normalization. (C) P values from linear models testing the relationship between each known variable (potential covariates and variable of interest) and the principal components that explain at least 5% of the total variance in the gene expression data are shown. P values that are significant (after Bonferroni correction at α = 0.05) are highlighted in red. (PDF) [file pgen.1005111.s001.pdf]

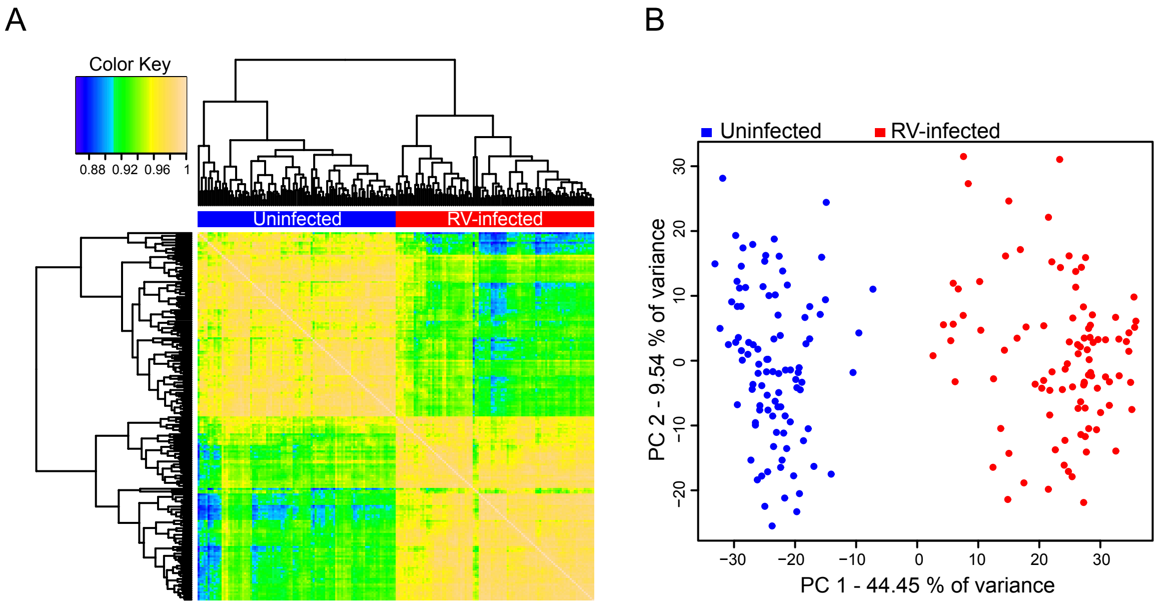

C

PC (Variance Explained)	Treatment	Gender	Virus Batch	Processing Day	Chip Number	PBMC count	Age	Ancestry Fraction 1	Ancestry Fraction 2	Ancestry Fraction 3
PC1 (44.45%)	<2.20E-16	0.3722	0.1438	0.6708	0.6359	0.2886	0.8437	0.8738	0.6851	0.6958
PC2 (9.54%)	0.4736	0.5948	0.02653	<2.20E-16	0.1665	0.0954	0.0028	0.1344	0.0023	0.00369
PC3 (7.22%)	0.02592	0.0007	<2.20E-16	<2.20E-16	0.0807	9.70E-08	0.0007	0.6815	0.5302	0.5565
PC4 (6.27%)	0.5003	0.988	7.47E-08	1.22E-15	0.652	0.2093	0.0262	0.06226	0.8927	0.7412
