# Supplementary material for: Host Genetic Variation Influences Gene Expression Response to Rhinovirus Infection
Source: PLoS Genet. 2015 Apr 13;11(4):e1005111. doi: 10.1371/journal.pgen.1005111 (PMC4395341; doi:10.1371/journal.pgen.1005111)
Supplement: S2 Fig — (A) Heatmap clustering, and (B) Principal component plot of 13,881 probes after log2-transformation, rank-invariate normalization, and regressing out processing day. (C) P values from linear models testing the relationship between each known variable (potential covariates and variable of interest) and the principal components that explain at least 5% of the total variance in the gene expression data are shown. P values that are significant (after Bonferroni correction at α = 0.05) are highlighted in red. (PDF) [file pgen.1005111.s002.pdf]

A

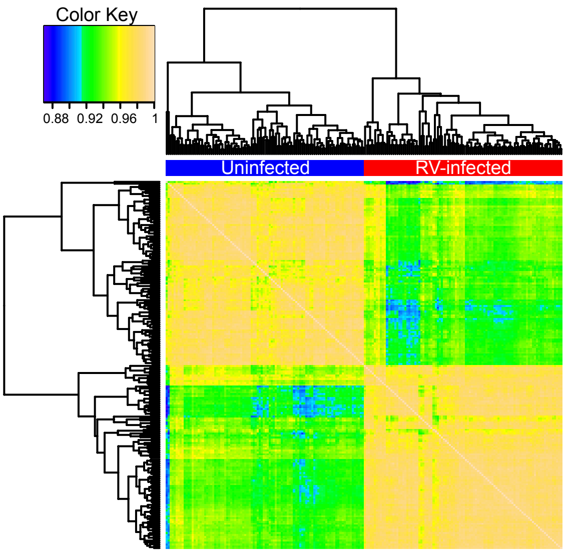

B

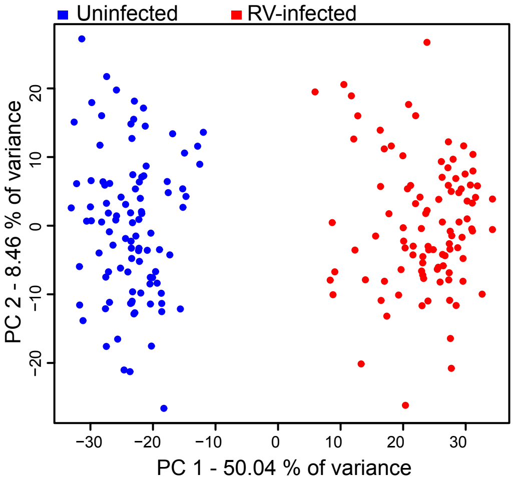

C

PC (Variance Explained)	Treatment	Gender	Virus Batch	Processing Day	Chip Number	PBMC count	Age	Ancestry Fraction 1	Ancestry Fraction 2	Ancestry Fraction 3
PC1 (50.04%)	<2.20E-16	0.4567	1	1	0.75	0.7836	0.7543	0.8245	0.7403	0.7215
PC2 (8.46%)	0.8569	0.302	1	1	0.4193	0.0519	0.0557	0.1185	0.1204	0.1624
PC3 (5.32%)	0.1946	0.0066	1	1	0.6354	0.1463	0.1272	0.9472	0.04747	0.04704
