# Supplementary figures and images for: Host Genetic Variation Influences Gene Expression Response to Rhinovirus Infection
Source: PLoS Genet. 2015 Apr 13;11(4):e1005111. doi: 10.1371/journal.pgen.1005111 (PMC4395341; doi:10.1371/journal.pgen.1005111)

**A**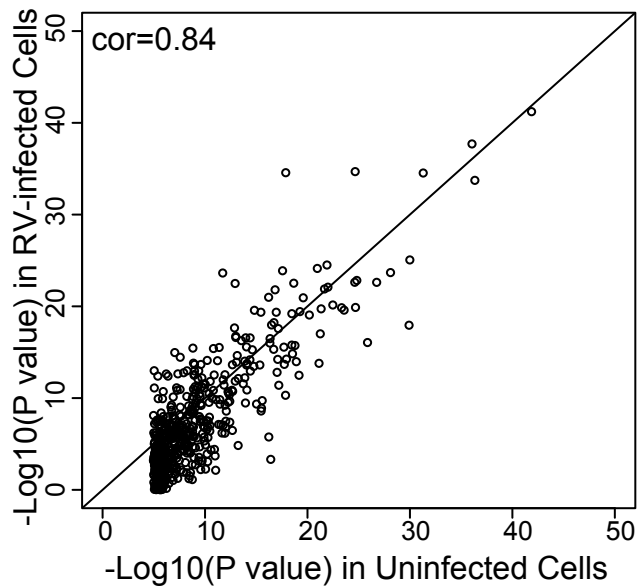**B**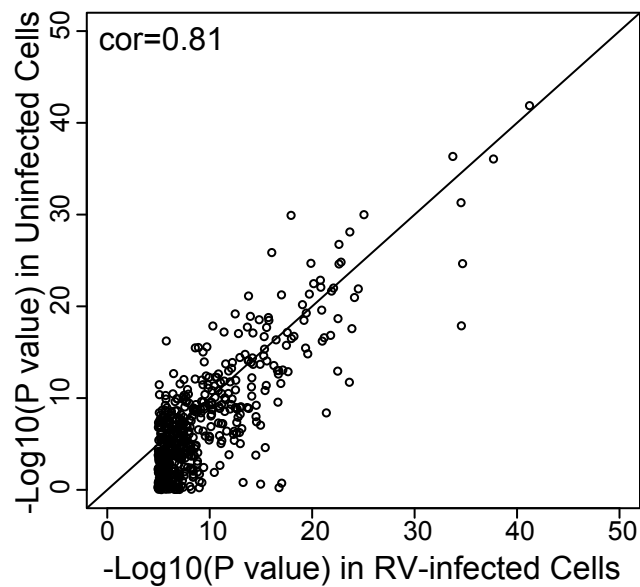**C**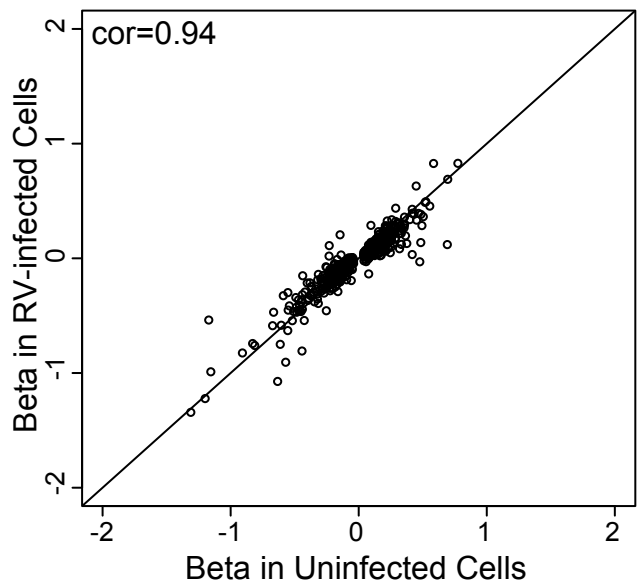**D**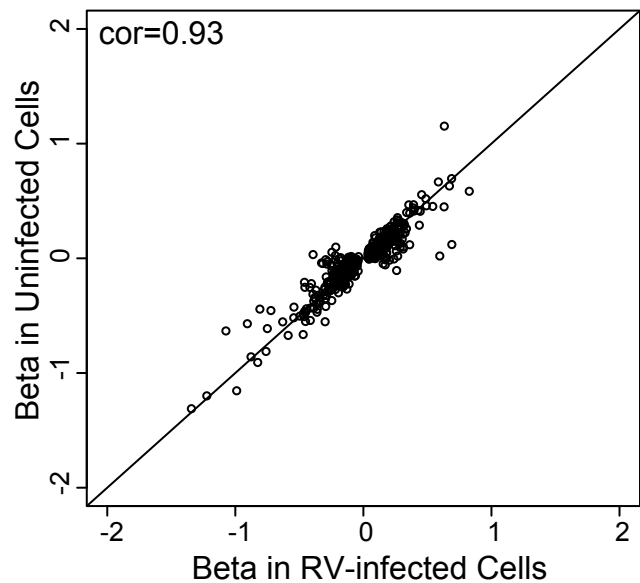

Supplement: S3 Fig — (A) For 521 significant local eQTL-gene expression pair in uninfected cells, P values in uninfected cells are shown on the x-axis and P values in RV-infected cells are shown on the y-axis. Pearson correlation of eQTL association P values was 0.84 (P<2.2x10^-16^). (B) For 523 significant local eQTL-gene expression pair in RV-infected cells, P values in RV-infected cells are shown on the x-axis and P values in uninfected cells are shown on the y-axis. Pearson correlation of eQTL association P values was 0.81 (P<2.2x10^-16^). (C) For 521 significant local eQTL-gene expression pair in uninfected cells, Beta (effect size estimate) values in uninfected cells are shown on the x-axis and Beta values in RV-infected cells are shown on the y-axis. Pearson correlation of Beta values was 0.94 (P<2.2x10^-16^). (D) For 523 significant local eQTL-gene expression pair in RV-infected cells, Beta values in RV-infected cells are shown on the x-axis and Beta values in uninfected cells are shown on the y-axis. Pearson correlation of Beta values was 0.93 (P<2.2x10^-16^). (PDF) [file pgen.1005111.s003.pdf]

## Genes with reQTLs

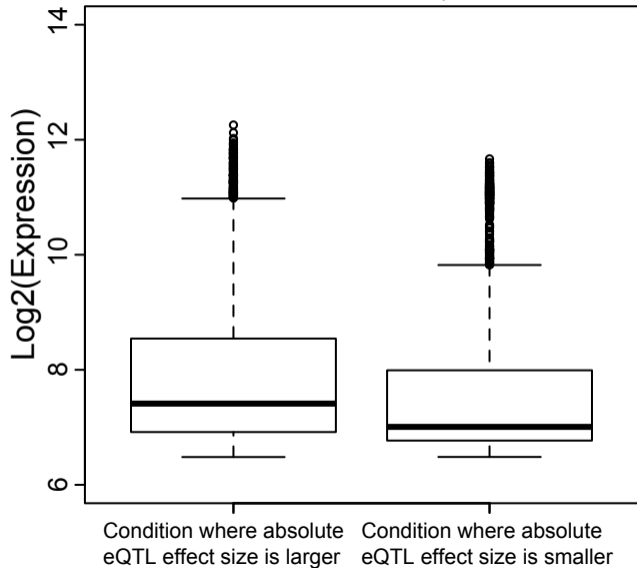

Supplement: S4 Fig — (PDF) [file pgen.1005111.s004.pdf]

**A**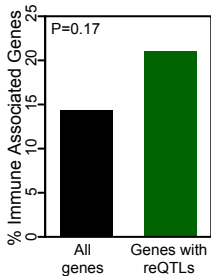**B**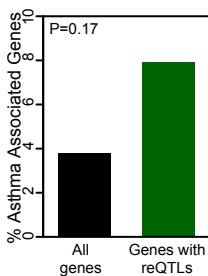**C**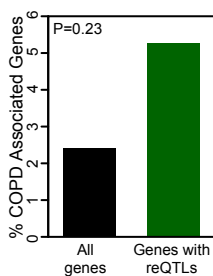**D**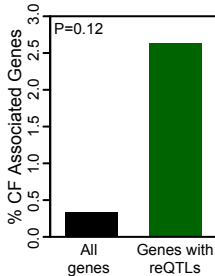

Supplement: S5 Fig — Percentage of genes associated with (A) Immune diseases (B) Asthma (C) Chronic Obstructive Pulmonary Disease (COPD) (D) Cystic Fibrosis (CF). In each panel, ‘all genes’ category (in black) refers to 10,893 genes detected as expressed and ‘genes with reQTLs’ category (in green) refers to 38 genes that had a significant local reQTL in our study. Enrichment P values (Fisher’s exact test) were 0.17, 0.17, 0.23, and 0.12, respectively. (PDF) [file pgen.1005111.s005.pdf]

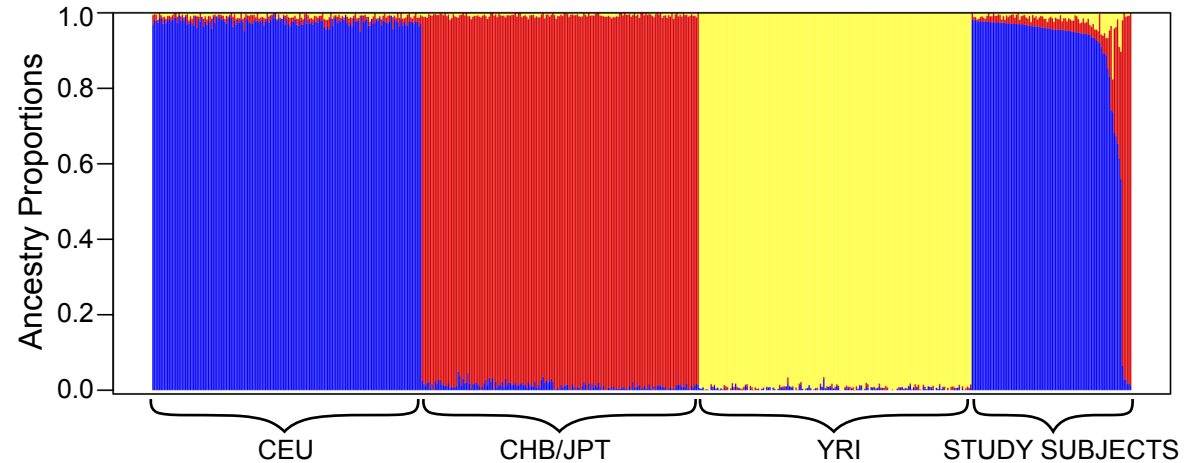

Supplement: S7 Fig — Subjects from the Phase 3 HapMap CEU (Utah residents with Northern and Western European ancestry from the CEPH collection), CHB (Han Chinese in Beijing, China), JPT (Japanese in Tokyo, Japan), and YRI (Yoruba in Ibadan, Nigeria) were included as reference. (PDF) [file pgen.1005111.s007.pdf]
