# Supplementary material for: Host Genetic Variation Influences Gene Expression Response to Rhinovirus Infection
Source: PLoS Genet. 2015 Apr 13;11(4):e1005111. doi: 10.1371/journal.pgen.1005111 (PMC4395341; doi:10.1371/journal.pgen.1005111)
Supplement: S6 Fig — (PDF) [file pgen.1005111.s006.pdf]

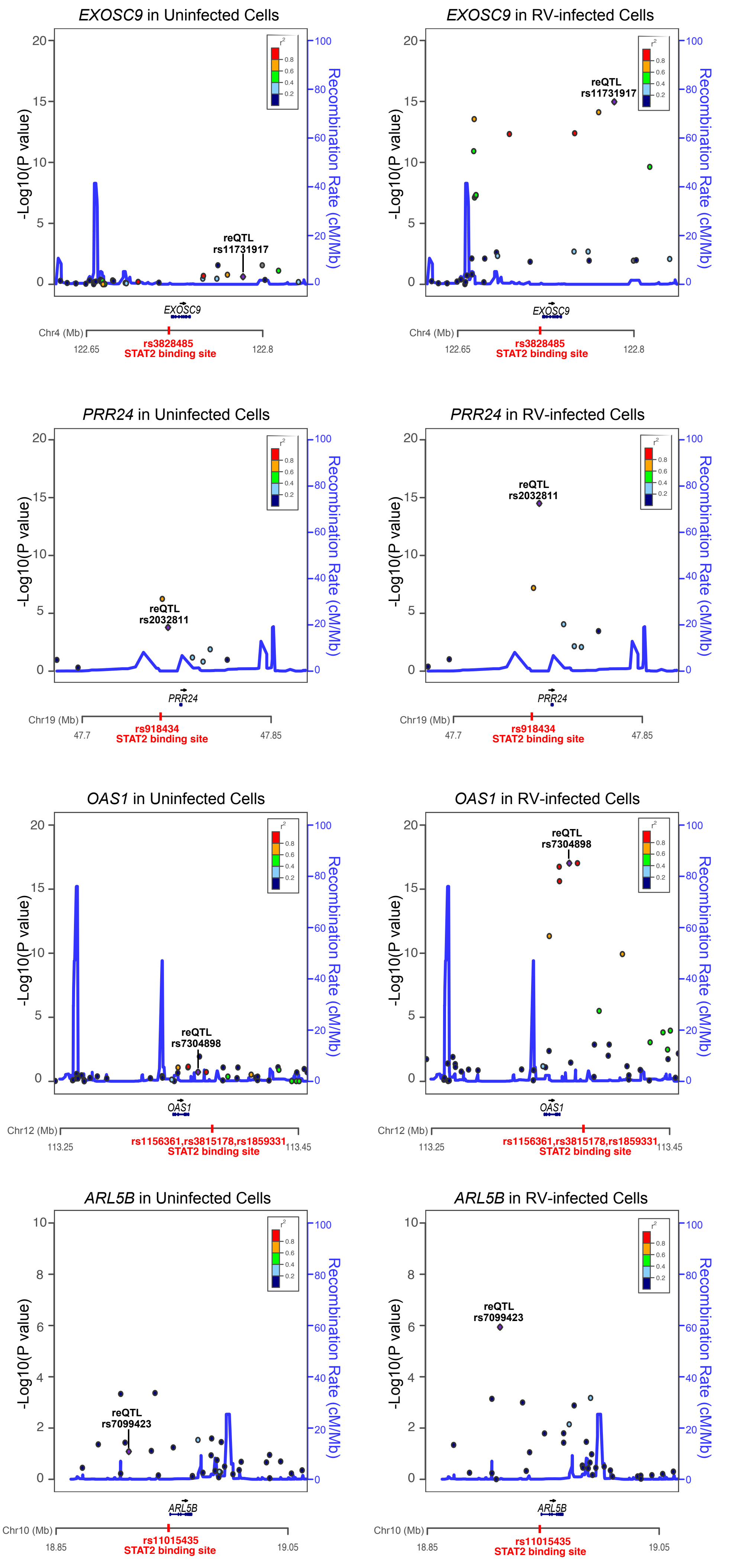
EXOSC9 in Uninfected Cells
EXOSC9 in RV-infected Cells
-Log10(P value)
Recombination Rate (cM/Mb)
r2
0.8
0.6
0.4
0.2
reQTL
rs11731917
EXOSC9
Chr4 (Mb)
122.65
122.8
rs3828485
STAT2 binding site
PRR24 in Uninfected Cells
PRR24 in RV-infected Cells
reQTL
rs2032811
PRR24
Chr19 (Mb)
47.7
47.85
rs918434
STAT2 binding site
OAS1 in Uninfected Cells
OAS1 in RV-infected Cells
reQTL
rs7304898
OAS1
Chr12 (Mb)
113.25
113.45
rs1156361,rs3815178,rs1859331
STAT2 binding site
ARL5B in Uninfected Cells
ARL5B in RV-infected Cells
reQTL
rs7099423
ARL5B
Chr10 (Mb)
18.85
19.05
rs11015435
STAT2 binding site
