# Supplementary material for: Host Genetic Variation Influences Gene Expression Response to Rhinovirus Infection
Source: PLoS Genet. 2015 Apr 13;11(4):e1005111. doi: 10.1371/journal.pgen.1005111 (PMC4395341; doi:10.1371/journal.pgen.1005111)
Supplement: S1 Table — Among the genes that were targeted in both studies, 86.5% (32 out of 37) with ≥2-fold increase in response to RV infection in BECs also showed ≥2-fold increase in response to RV infection in PBMCs. (PDF) [file pgen.1005111.s008.pdf]

Table S1. Overlap between RV-responsive genes in bronchial epithelial cells (BECs) and in PBMCs. Among the genes that were targeted in both studies, 86.5% (32 out of 37) with ≥2-fold increase in response to RV infection in BECs also showed ≥2-fold increase in response to RV infection in PBMCs.

Gene Name	Fold Increase in BECs	Fold Increase in PBMCs
CXCL10	11.5	25.39
C1orf29/IFI44L	16.17	25.02
ISG15	13.86	21.18
IFIT4/IFIT3	4.36	21.00
Viperin/RSAD2	11.51	20.92
IFIT2	4.11	18.42
IFITM3	2.05	16.48
OAS1	4.88	12.27
IFI44	3.84	11.71
IFIT1	21.47	11.40
MX2	9.01	11.04
MX1	9.94	10.51
LOC51191/HERC5	3.05	10.25
OASL	2.61	9.86
IRF7	5.5	8.27
G1P3/IFI6	8.13	7.82
HSXIAPAF1/XAF1	2.79	7.28
IFI35	4.09	6.92
GBP1	4.16	6.48
OAS2	6.05	5.59
LAMP3	6.06	5.32
ISG20	2.82	5.11
IFITM2	2.1	4.99
RIG-I/DDX58	3.79	4.98
OAS3	6.19	4.13
SP110	2.29	4.05
LAP3	2.69	3.63
ECGF1/TYMP	2.2	3.24
BST2	5.16	3.17
WARS	4.13	3.16
STAT1	4.08	2.94
TOR1B	2.27	2.57
APOBEC3A	2.37	1.45
TRIM14	2.19	1.23
HRASLS2	2.3	1.11
CIC	2.53	1.02
CXCL11	17.64	Not expressed
FLJ20637	3.43	Not targeted / Excluded during probe QC
MDA5	3.5	Not targeted / Excluded during probe QC
FLJ20073	2.65	Not targeted / Excluded during probe QC
FLJ22693	2.57	Not targeted / Excluded during probe QC
FLJ20035	2.45	Not targeted / Excluded during probe QC

Continued on next page

Table S1 – Continued from previous page

PKR	2.3	Not targeted / Excluded during probe QC
ISGF3G	2.12	Not targeted / Excluded during probe QC
IFRG28	2.02	Not targeted / Excluded during probe QC
IFI27	12.44	Not targeted / Excluded during probe QC
IFITM1	4.63	Not targeted / Excluded during probe QC
USP18	2.44	Not targeted / Excluded during probe QC
