# Supplementary material for: Host Genetic Variation Influences Gene Expression Response to Rhinovirus Infection
Source: PLoS Genet. 2015 Apr 13;11(4):e1005111. doi: 10.1371/journal.pgen.1005111 (PMC4395341; doi:10.1371/journal.pgen.1005111)
Supplement: S2 Table — Among the genes that were targeted in both studies, 73.3% (22 out of 30) with ≥2-fold increase in response to RV infection in NESs also showed ≥2-fold increase in response to RV infection in PBMCs. (PDF) [file pgen.1005111.s009.pdf]

Table S2. Overlap between RV-responsive genes in nasal epithelial scrapings (NESs) and in PBMCs. Among the genes that were targeted in both studies, 73.3% (22 out of 30) with ≥2-fold increase in response to RV infection in NESs also showed ≥2-fold increase in response to RV infection in PBMCs.

Gene Name	Fold Increase in NESs	Fold Increase in PBMCs
CCL8	43.8	71.45
CXCL10	25.5	25.39
G1P2/ISG15	7.61	21.18
IFIT4/IFIT3	7.56	21.00
CIG5/RSAD2	6.72	20.92
IFIT2	11.4	18.42
CCL2	55	16.57
OAS1	3.78	12.27
IFIT1	8.82	11.40
MX2	4.26	11.04
MX1	3.29	10.51
OASL	4.57	9.86
IRF7	4.16	8.27
CXCL9	12.9	8.07
EPSTI1	4.45	7.66
GBP1	11.1	6.48
OAS2	4.35	5.59
RIGI/DDX58	3.45	4.98
PLSCR1	2.53	4.17
OAS3	4.72	4.13
SOCS1	31.7	3.96
HAPLN3	6.43	3.47
SOCS2	6.93	1.79
CCL20	12	1.50
ICAM-1/ICAM1	8.48	1.31
CXCL13	14.8	1.21
SOCS3	2.91	1.08
TNC	2.8	0.97
CXCL11	37.4	Not expressed
MMP12	4.7	Not expressed
NOS2A	6.07	Not targeted / Excluded during probe QC
IL28B/IFNL3	5.56	Not targeted / Excluded during probe QC
MDA5	3.69	Not targeted / Excluded during probe QC
