# Supplementary material for: Host Genetic Variation Influences Gene Expression Response to Rhinovirus Infection
Source: PLoS Genet. 2015 Apr 13;11(4):e1005111. doi: 10.1371/journal.pgen.1005111 (PMC4395341; doi:10.1371/journal.pgen.1005111)
Supplement: S3 Table — Enriched GO categories at P value cut-off of 10–5 are shown. (PDF) [file pgen.1005111.s010.pdf]

**Table S3.** Gene ontology (GO) enrichment analysis for the 271 genes that were both statistically differentially expressed and had a fold change of ≥2 in response to RV infection. Enriched GO categories at P value cut-off of $10^{-5}$ are shown.

| Enriched GO Categories | Observed Number of Genes | Expected Number of Genes | Fold Enrichment | Enrichment P Value |
|---|---|---|---|---|
| response to stimulus | 122 | 50.83 | 2.4 | $1.20x10^{-25}$ |
| response to other organism | 40 | 4.94 | 8.1 | $4.00x10^{-25}$ |
| response to biotic stimulus | 43 | 6.62 | 6.5 | $5.50x10^{-23}$ |
| immune system process | 67 | 17.63 | 3.8 | $9.30x10^{-23}$ |
| immune response | 54 | 11.74 | 4.6 | $1.10x10^{-21}$ |
| response to virus | 27 | 2.23 | 12.1 | $1.10x10^{-21}$ |
| defense response | 49 | 10.00 | 4.9 | $6.70x10^{-21}$ |
| response to external stimulus | 54 | 14.21 | 3.8 | $9.50x10^{-18}$ |
| multi-organism process | 49 | 12.25 | 4 | $5.80x10^{-17}$ |
| response to stress | 75 | 28.85 | 2.6 | $2.80x10^{-15}$ |
| response to wounding | 36 | 8.51 | 4.2 | $3.70x10^{-13}$ |
| inflammatory response | 27 | 5.40 | 5 | $1.60x10^{-11}$ |
| response to chemical stimulus | 57 | 21.92 | 2.6 | $1.60x10^{-11}$ |
| taxis | 18 | 2.73 | 6.6 | $9.80x10^{-10}$ |
| chemotaxis | 18 | 2.73 | 6.6 | $9.80x10^{-10}$ |
| response to molecule of bacterial origin | 13 | 1.78 | 7.3 | $1.30x10^{-07}$ |
| response to lipopolysaccharide | 12 | 1.58 | 7.6 | $2.90x10^{-07}$ |
| locomotory behavior | 18 | 4.09 | 4.4 | $5.10x10^{-07}$ |
| locomotion | 22 | 6.29 | 3.5 | $8.40x10^{-07}$ |
| regulation of defense response | 14 | 2.59 | 5.4 | $1.30x10^{-06}$ |
| response to bacterium | 15 | 3.00 | 5 | $1.30x10^{-06}$ |
| response to organic substance | 32 | 12.80 | 2.5 | $2.40x10^{-06}$ |
| innate immune response | 13 | 2.32 | 5.6 | $2.70x10^{-06}$ |
| cell-cell signaling | 22 | 6.67 | 3.3 | $2.90x10^{-06}$ |
| regulation of response to stimulus | 25 | 8.62 | 2.9 | $3.30x10^{-06}$ |
| regulation of cytokine production | 16 | 3.72 | 4.3 | $3.70x10^{-06}$ |
| cell communication | 28 | 10.37 | 2.7 | $4.20x10^{-06}$ |
| cellular di-, tri-valent inorganic cation homeostasis | 15 | 3.41 | 4.4 | $6.50x10^{-06}$ |
