# Supplementary material for: Host Genetic Variation Influences Gene Expression Response to Rhinovirus Infection
Source: PLoS Genet. 2015 Apr 13;11(4):e1005111. doi: 10.1371/journal.pgen.1005111 (PMC4395341; doi:10.1371/journal.pgen.1005111)
Supplement: S4 Table — Enriched KEGG pathways at P value cut-off of 0.05 are shown. (PDF) [file pgen.1005111.s011.pdf]

Table S4. Kyoto Encyclopedia of Genes and Genomes (KEGG) pathway enrichment analysis for the 271 genes that were both statistically differentially expressed and had a fold change of ≥2 in response to RV infection. Enriched KEGG pathways at P value cut-off of 0.05 are shown.

Enriched KEGG Pathways	Observed Number of Genes	Expected Number of Genes	Fold Enrichment	Enrichment P Value
Cytosolic DNA-sensing pathway	14	1.87	7.5	$1.20x10^{-08}$
Toll-like receptor signaling pathway	16	3.33	4.8	$5.20x10^{-07}$
Cytokine-cytokine receptor interaction	21	6.00	3.5	$9.50x10^{-07}$
RIG-I-like receptor signaling pathway	13	2.32	5.6	$1.70x10^{-06}$
Autoimmune thyroid disease	8	0.98	8.2	$2.80x10^{-05}$
Antigen processing and presentation	10	1.96	5.1	$9.10x10^{-05}$
Chemokine signaling pathway	14	5.19	2.7	$1.70x10^{-03}$
Regulation of autophagy	6	0.94	6.4	$1.80x10^{-03}$
Jak-STAT signaling pathway	12	4.14	2.9	$2.00x10^{-03}$
PPAR signaling pathway	6	1.40	4.3	$1.10x10^{-02}$
Natural killer cell mediated cytotoxicity	9	3.75	2.4	$3.00x10^{-02}$
Arachidonic acid metabolism	4	0.85	4.7	$4.90x10^{-02}$
