# Supplementary material for: Host Genetic Variation Influences Gene Expression Response to Rhinovirus Infection
Source: PLoS Genet. 2015 Apr 13;11(4):e1005111. doi: 10.1371/journal.pgen.1005111 (PMC4395341; doi:10.1371/journal.pgen.1005111)
Supplement: S5 Table — (PDF) [file pgen.1005111.s012.pdf]

Table S5. Comparison of most significant reQTL based on imputed and genotyped data.

Hugo Gene ID	Ensembl Gene ID	Most Significant Local reQTL Based on Genotyped Data	P value reQTL Based on Genotyped Data	Most Significant Local reQTL Based on Imputed Data	P value reQTL Based on Imputed Data
EXOSC9	ENSG00000123737	rs11731917	3.95E-17	rs55797638	2.91E-18
IRF5	ENSG00000128604	rs7807018	9.12E-18	rs10954213<br>rs13242262<br>rs10229001<br>rs4731533<br>rs4731534<br>rs2272347<br>rs7796963<br>rs6969930<br>rs11763684<br>rs7807018<br>rs4731536<br>rs10954215<br>rs11767711<br>rs6965542<br>rs12155080<br>rs60086303<br>rs10239340<br>rs34842192	9.12E-18
SLFN5	ENSG00000166750	rs11867191	4.73E-15	rs883416	4.72E-15
ANKDD1A	ENSG00000166839	rs871447	2.35E-14	rs28605697<br>rs9920138<br>rs832891<br>rs871447	2.35E-14
PRR24	ENSG00000257704	rs2032811	5.38E-14	rs2032811	2.60E-14
UBA7	ENSG00000182179	rs7649348	1.51E-12	rs7649348	1.51E-12
TMTC1	ENSG00000133687	rs4931220	2.31E-11	12:29912275	1.93E-11
MASTL	ENSG00000120539	rs11598615	4.81E-10	rs7080173<br>rs7922972	4.86E-11
FBN2	ENSG00000138829	rs6884224	7.50E-10	rs71955823	1.03E-10
AGTRAP	ENSG00000177674	rs7414943	5.22E-10	rs7414943	5.22E-10
MYO1D	ENSG00000176658	rs17182914	7.85E-10	rs17182935	6.41E-10
GSTM3	ENSG00000134202	rs10735234	1.89E-09	rs1109138<br>rs5776997<br>rs10735234	1.89E-09
KCNF1	ENSG00000162975	rs62120322	7.09E-07	rs1574302	3.86E-09
STOX1	ENSG00000165730	rs3740593	2.36E-08	rs35949960<br>rs12771648<br>rs150327559<br>rs61869906	4.63E-09
TMEM176A	ENSG00000002933	rs6464100	1.37E-08	rs4725942<br>rs4725364<br>rs9691673	5.90E-09
IL36RN	ENSG00000136695	rs315946	6.82E-09	rs315946	6.82E-09
HOMER2	ENSG00000103942	rs10520576	1.53E-08	rs62010177	6.83E-09

Continued on next page

Table S5 – Continued from previous page

SLC35F3	ENSG00000183780	rs594657	9.86E-08	rs35822885	1.36E-08
SPTLC2	ENSG00000100596	rs11159275	1.43E-08	rs2012998<br>rs2003633<br>rs60221235<br>rs36063635<br>rs8003639<br>rs8003812<br>rs11159275<br>rs741850<br>rs2886131	1.43E-08
RUFY4	ENSG00000188282	rs10187555	1.75E-07	rs111856394<br>rs114593680	2.16E-08
AMDHD1	ENSG00000139344	rs7486703	3.38E-08	12:96337183<br>rs7486703	3.38E-08
DNTTIP1	ENSG00000101457	rs6124742	1.32E-07	rs459955<br>rs380421	3.57E-08
OAS1	ENSG00000089127	rs7304898	2.59E-07	12:113370427<br>12:113374748	4.09E-08
ARL5B	ENSG00000165997	rs7099423	3.34E-07	rs3740104	4.15E-08
MFAP5	ENSG00000197614	rs7132012	4.27E-08	rs7132012	4.27E-08
ZSWIM7	ENSG00000214941	rs4792715	2.42E-07	rs34243080	7.64E-08
MSR1	ENSG00000038945	rs1405286	1.24E-07	rs68112997	9.74E-08
CCDC146	ENSG00000135205	rs17544690	1.21E-07	rs67414486	1.12E-07
KIAA0664L3	ENSG00000131797	rs1534507	3.26E-07	rs1045483<br>rs34997130	1.45E-07
PODXL	ENSG00000128567	rs6467369	1.74E-07	7:130978775<br>rs6467369	1.74E-07
INPP1	ENSG00000151689	rs10931450	3.29E-07	rs10188870	1.80E-07
CALM1	ENSG00000198668	rs2343	3.14E-07	rs1107073	2.29E-07
ADCY3	ENSG00000138031	rs2033655	5.51E-07	rs1541984	3.27E-07
ITGA2	ENSG00000164171	rs17298242	6.53E-07	rs17298242	6.53E-07
PDE1B	ENSG00000123360	rs3782404	6.60E-07	rs3782404	6.60E-07
RAB31	ENSG00000168461	KG_18_9718160	6.67E-07	KG_18_9718160	6.67E-07
GJA3	ENSG00000121743	rs7981775	6.91E-07	rs7981775	6.91E-07
C17orf97	ENSG00000187624	rs62056208	4.44E-07	NA	NA
