# Supplementary material for: Host Genetic Variation Influences Gene Expression Response to Rhinovirus Infection
Source: PLoS Genet. 2015 Apr 13;11(4):e1005111. doi: 10.1371/journal.pgen.1005111 (PMC4395341; doi:10.1371/journal.pgen.1005111)
Supplement: S6 Table — (PDF) [file pgen.1005111.s013.pdf]

Table S6. ChIP-Seq enrichment results of input SNPs relative to the background SNPs based on all available human cell types and treatment conditions in the ENCODE data.

Protein	Observed Number of SNPs Overlapping with ChIP-Seq annotation	Expected Number of SNPs Overlapping with ChIP-Seq annotation	Fold Enrichment	Enrichment P value
STAT2	7	0.43	16.30	3.63E-07
SP2	4	0.67	6.01	0.004827995
E2F4	10	1.77	5.65	1.64E-05
HMGN3	13	3.24	4.02	3.37E-05
SP1	25	6.25	4.00	1.15E-08
SUZ12	4	1.01	3.96	0.019493751
ETS1	9	2.31	3.90	0.000650801
THAP1	2	0.53	3.76	0.100042713
MEF2C	3	0.85	3.52	0.055019162
RXRA	8	2.30	3.48	0.002573065
POL2S2	9	3.01	2.99	0.003880327
BRCA1	3	1.01	2.96	0.08261176
SIN3AK20	14	4.86	2.88	0.000513032
BCL3	4	1.39	2.88	0.052207273
STAT1	7	2.45	2.86	0.012695208
NRF1	5	1.88	2.67	0.042038959
PU1	19	7.13	2.66	0.000156385
CCNT2	11	4.18	2.63	0.003879436
TCF4	13	5.00	2.60	0.001966539
IRF1	8	3.11	2.57	0.014338596
ELF1	19	7.63	2.49	0.000353747
HAE2F1	18	7.27	2.48	0.000536636
SRF	5	2.04	2.45	0.056335103
TCF12	7	2.88	2.43	0.027888196
TAF1	22	9.54	2.31	0.000361381
HEY1	16	7.04	2.27	0.002468287
ELK4	4	1.77	2.26	0.103737791
NFYB	4	1.77	2.26	0.104358622
IRF4	5	2.23	2.24	0.075691726
EBF1	12	5.41	2.22	0.00958252
NFKB	17	7.70	2.21	0.002492332
POL24H8	30	14.03	2.14	0.000128488
ATF3	2	0.95	2.11	0.245770054
CFOS	16	7.83	2.04	0.006687411
RFX5	6	2.96	2.03	0.079697216
STAT3	14	7.08	1.98	0.013728924

Continued on next page

Table S6 – Continued from previous page

CEBPB	22	11.23	1.96	0.002759492
ZBTB7A	7	3.60	1.95	0.072864369
PAX5C20	8	4.17	1.92	0.061858545
E2F1	3	1.60	1.88	0.215577711
MEF2A	4	2.19	1.83	0.17879643
YY1	16	8.90	1.80	0.019852466
SIX5	2	1.12	1.79	0.308030207
POL2	51	29.31	1.74	0.00014804
CJUN	10	5.97	1.68	0.081210134
BAF155	2	1.19	1.67	0.335279941
SETDB1	8	4.82	1.66	0.114500558
P300	18	10.97	1.64	0.030880187
TAF7	3	1.83	1.64	0.278087048
POL2B	7	4.28	1.63	0.141983637
FOXA2	6	3.75	1.60	0.177141182
CHD2	4	2.52	1.59	0.24625173
NFYA	3	1.94	1.55	0.305735142
E2F6	8	5.24	1.53	0.158959732
FOSL2	4	2.63	1.52	0.271032716
ZNF143	4	2.75	1.46	0.296215796
GATA3	5	3.61	1.39	0.295372892
USF2	4	2.90	1.38	0.331367928
HNF4G	3	2.23	1.35	0.3844789
POU2F2	4	3.03	1.32	0.359640876
OCT2	4	3.05	1.31	0.364942853
USF1	10	8.08	1.24	0.292899924
BATF	3	2.47	1.22	0.447821673
HNF4A	4	3.29	1.22	0.417694026
GABP	5	4.11	1.22	0.39353891
CTCF	28	23.15	1.21	0.17936741
TBP	11	9.16	1.20	0.313156146
AP2GAMMA	5	4.32	1.16	0.432634702
JUND	11	9.52	1.16	0.357125172
MAFF	4	3.50	1.14	0.464273736
GATA1	6	5.32	1.13	0.440175825
MXI1	3	2.71	1.11	0.509338766
CMYC	11	10.05	1.10	0.42269294
MAX	11	10.06	1.09	0.424177322
NRSF	4	3.83	1.05	0.532200012
EGR1	6	6.12	0.98	0.573849446
INI1	2	2.05	0.97	0.608426063

Continued on next page

Table S6 – Continued from previous page

GATA2	9	9.80	0.92	0.645115619
SMC3	5	5.75	0.87	0.680033696
FOXA1	8	9.28	0.86	0.70838594
RAD21	9	11.88	0.76	0.838231855
MAFK	5	7.35	0.68	0.857611967
AP2ALPHA	2	3.01	0.67	0.802217129
TAL1	2	3.13	0.64	0.819119374
GR	2	3.67	0.55	0.881269064
ZNF263	3	6.83	0.44	0.966718443
