# Supplementary material for: Host Genetic Variation Influences Gene Expression Response to Rhinovirus Infection
Source: PLoS Genet. 2015 Apr 13;11(4):e1005111. doi: 10.1371/journal.pgen.1005111 (PMC4395341; doi:10.1371/journal.pgen.1005111)
Supplement: S7 Table — LD (r2) is based on 1000 Genomes Phase 1 EUR population. (PDF) [file pgen.1005111.s014.pdf]

Table S7. reQTL loci variants that overlap with STAT2 ChIP-Seq annotation. LD (r^2) is based on 1000 Genomes Phase 1 EUR population.

Hugo Gene ID	Ensembl Gene ID	Most Significant Local reQTL Based on Genotyped Data	Variant that Overlaps with STAT2 ChIP-Seq Annotation	Cell Type/Treatment in which ChIP-Seq Signal was Annotated	LD (r^2) Between Most Significant Local reQTL and Variant that Overlaps with STAT2 binding site
EXOSC9	ENSG00000123737	rs11731917	rs3828485	K562/IFNa30min, K562/IFNa6h	0.92
ARL5B	ENSG00000165997	rs7099423	rs11015435	K562/IFNa6h	0.87
OAS1	ENSG00000089127	rs7304898	rs1156361	K562/IFNa6h	0.98
OAS1	ENSG00000089127	rs7304898	rs3815178	K562/IFNa6h	0.97
OAS1	ENSG00000089127	rs7304898	rs1859331	K562/IFNa6h	0.95
SLFN5	ENSG00000166750	rs11867191	rs11080327	K562/IFNa30min, K562/IFNa6h	0.95
PRR24	ENSG00000257704	rs2032811	rs918434	K562/IFNa30min	0.94
