# Supplementary material for: Host Genetic Variation Influences Gene Expression Response to Rhinovirus Infection
Source: PLoS Genet. 2015 Apr 13;11(4):e1005111. doi: 10.1371/journal.pgen.1005111 (PMC4395341; doi:10.1371/journal.pgen.1005111)
Supplement: S8 Table — (PDF) [file pgen.1005111.s015.pdf]

Table S8. Quality control check of genome-wide significant distant eQTLs **A)** in uninfected cells **B)** in RV-infected cells. For each gene that was putatively *trans* regulated, we attempted to map each of its probes within 2 Mb of the *trans*-eQTL using SHRiMP with relaxed mapping setting; match score of 10, mismatch score of 0, gap open penalty of −250, gap extension penalty of −100, and minimal Smith-Waterman score of 30%. T*rans*-eQTLs whose associated trans-probe mapped its vicinity failed quality control check.

A)

Hugo Gene ID	Ensembl Gene ID	Probe ID	Trans eQTL	Trans eQTL Chr Position	Target Locus		Potential Cross-Hybridization Locus		Passed QC
					Alignment Identity	Chr Position	Alignment Identity	Chr Position	
RP11-824M15.1	ENSG00000224161	ILMN_3291511	rs10876864	chr12:56401085	100%	18:57428812-57428861	90%	12:56436228-56436277	No
RP11-824M15.1	ENSG00000224161	ILMN_1737991	rs10876864	chr12:56401085	100%	18:57429067-57429116	80%	12:56437243-56437292	No
RP11-459K23.1	ENSG00000227887	ILMN_3236675	rs10876864	chr12:56401085	100%	1:208870720-208870769	72%	12:56437240-56437289	No
RPS26P2	ENSG00000233278	ILMN_3290019	rs10876864	chr12:56401085	100%	9:30831910-30831959	94%	12:56437230-56437279	No
RP11-777J24.1	ENSG00000233778	ILMN_1657950	rs10876864	chr12:56401085	100%	8:93156573-93156622	90%	12:56436247-56436296	No
RP11-57C13.5	ENSG00000234192	ILMN_3190596	rs10876864	chr12:56401085	100%	10:89402626-89402675	92%	12:56437228-56437277	No
RPS26P35	ENSG00000242206	ILMN_1677697	rs10876864	chr12:56401085	100%	8:119774202-119774251	96%	12:56437153-56437202	No
RP11-713H12.1	ENSG00000244604	ILMN_3296994	rs10876864	chr12:56401085	100%	17:8464670-8464719	72%	12:56436328-56436377	No
SBNO2	ENSG00000064932	ILMN_1808811	rs890294	chr5:94405027	100%	19:1107866-1107915			Yes
STX1B	ENSG00000099365	ILMN_3234081	rs10876864	chr12:56401085	100%	16:31000580-31000629			Yes
STX1B	ENSG00000099365	ILMN_3189972	rs10876864	chr12:56401085	100%	16:31001369-31001418			Yes
STX1B	ENSG00000099365	ILMN_1660721	rs10876864	chr12:56401085	100%	16:31003560-31003609			Yes
CTD-2588C8.1	ENSG00000239210	ILMN_3254492	rs10876864	chr12:56401085	100%	19:35024337-35024386			Yes
GAPDHP61	ENSG00000248415	ILMN_3267451	rs2072373	chr12:6631888	100%	15:64820974-64821023			Yes

B)

Hugo Gene ID	Ensembl Gene ID	Probe ID	Trans eQTL	Trans eQTL Chr Position	Target Locus		Potential Cross-Hybridization Locus		Passed QC
					Alignment Identity	Chr Position	Alignment Identity	Chr Position	
RP11-824M15.1	ENSG00000224161	ILMN_3291511	rs10876864	chr12:56401085	100%	18:57428812-57428861	90%	12:56436228-56436277	No
RP11-824M15.1	ENSG00000224161	ILMN_1737991	rs10876864	chr12:56401085	100%	18:57429067-57429116	80%	12:56437243-56437292	No
RP11-196I18.3	ENSG00000225531	ILMN_3180196	rs2956114	chr11:34937813	100%	9:109879221-109879270	86%	11:34916554-34916603	No
RP11-196I18.3	ENSG00000225531	ILMN_3260618	rs2956114	chr11:34937813	100%	9:109879456-109879505	94%	11:34909905-34909954	No
RP11-459K23.1	ENSG00000227887	ILMN_3236675	rs10876864	chr12:56401085	100%	1:208870720-208870769	72%	12:56437240-56437289	No
RPS26P2	ENSG00000233278	ILMN_3290019	rs10876864	chr12:56401085	100%	9:30831910-30831959	94%	12:56437230-56437279	No
RP11-777J24.1	ENSG00000233778	ILMN_1657950	rs10876864	chr12:56401085	100%	8:93156573-93156622	90%	12:56436247-56436296	No
RP11-57C13.5	ENSG00000234192	ILMN_3190596	rs10876864	chr12:56401085	100%	10:89402626-89402675	92%	12:56437228-56437277	No
RP11-310H4.5	ENSG00000234844	ILMN_3282321	rs2268179	chr1:22414785	100%	7:55706083-55706132	96%	1:22413280-22413329	No
RPS26P35	ENSG00000242206	ILMN_1677697	rs10876864	chr12:56401085	100%	8:119774202-119774251	96%	12:56437153-56437202	No
RP11-713H12.1	ENSG00000244604	ILMN_3296994	rs10876864	chr12:56401085	100%	17:8464670-8464719	72%	12:56436328-56436377	No
STX1B	ENSG00000099365	ILMN_3234081	rs10876864	chr12:56401085	100%	16:31000580-31000629			Yes
STX1B	ENSG00000099365	ILMN_3189972	rs10876864	chr12:56401085	100%	16:31001369-31001418			Yes
STX1B	ENSG00000099365	ILMN_1660721	rs10876864	chr12:56401085	100%	16:31003560-31003609			Yes
LIMS1	ENSG00000169756	ILMN_1675387	rs73727334	chr6:32570667	100%	2:109292339-109292388			Yes
LIMS1	ENSG00000169756	ILMN_2381037	rs73727334	chr6:32570667	100%	2:109300819-109300868			Yes
OR2AG1	ENSG00000170803	ILMN_1732467	rs1131964	chr21:34610487	100%	11:6806736-6806785			Yes
CTD-2588C8.1	ENSG00000239210	ILMN_3254492	rs10876864	chr12:56401085	100%	19:35024337-35024386			Yes
HBB	ENSG00000244734	ILMN_2100437	rs6124072	chr20:59759789	100%	11:5246808-5246857			Yes
GAPDHP61	ENSG00000248415	ILMN_3267451	rs11064213	chr12:6580376	100%	15:64820974-64821023			Yes
